# Longitudinal changes in the transcriptionally active and intact HIV reservoir after starting ART during acute infection

**DOI:** 10.1128/jvi.01431-24

**Published:** 2025-02-05

**Authors:** Julie Janssens, Adam Wedrychowski, Sun Jin Kim, Cordelia Isbell, Rebecca Hoh, Satish K. Pillai, Timothy J. Henrich, Steven G. Deeks, Nadia R. Roan, Sulggi A. Lee, Steven A. Yukl

**Affiliations:** 1 Department of Medicine, University of California, San Francisco (UCSF) 166668 , San Francisco, California, USA; 2 Department of Medicine, San Francisco Veterans Affairs Medical Center 19980 , San Francisco, California, USA; 3 Vitalant Research Institute 166672 , San Francisco, California, USA; 4 Department of Urology, University of California, San Francisco (UCSF) 360599 , San Francisco, California, USA; 5 Gladstone Institutes 40292 , San Francisco, California, USA; Icahn School of Medicine at Mount Sinai, New York, New York, USA

**Keywords:** human immunodeficiency virus, HIV-associated immune activation/inflammation, HIV persistence/latency

## Abstract

Even in antiretroviral therapy (ART)-suppressed human immunodeficiency virus (HIV)-infected individuals, there are heterogeneous populations of HIV-expressing cells exhibiting variable degrees of progression through blocks to HIV transcriptional initiation, elongation, completion, and splicing. These HIV-transcribing cells likely contribute to HIV-associated immune activation and inflammation as well as the viral rebound that occurs after stopping ART. However, it is unclear whether the blocks to HIV transcription are present before ART and how the timing and duration of ART may affect the clearance of cells expressing HIV transcripts that differ in their processivity and/or presence of mutations. To investigate these questions, we quantified different types of HIV transcripts and the corresponding HIV DNA regions/proviruses in longitudinal blood samples obtained before ART initiation (T1) and after 6 months (T2) and 1 year (T3) of ART in 16 individuals who initiated ART during acute HIV infection. Before ART, the pattern of HIV transcripts suggested blocks to elongation and splicing, and only ~10% of intact proviruses were transcribing intact HIV RNA. During the first 6 months of ART, we detected progressively greater reductions in initiated, 5′-elongated, mid-transcribed, completed, and multiply spliced HIV transcripts. Completed HIV RNA decayed faster than initiated or 5′-elongated HIV RNA, and intact HIV RNA tended to decay faster than defective HIV RNA. HIV DNA and RNA levels at T1–T3 correlated inversely with baseline CD4+ T-cell counts. Our findings suggest the existence of immune responses that act selectively to reduce HIV transcriptional completion and/or preferentially kill cells making completed or intact HIV RNA.

**IMPORTANCE:**

Even in virologically suppressed HIV-infected individuals, expression of viral products from both intact and defective proviruses may contribute to HIV-associated immune activation and inflammation, which are thought to underlie the organ damage that persists despite suppressive ART. We investigated how the timing of ART initiation and the duration of ART affect the heterogeneous populations of HIV-transcribing cells, including a detailed characterization of the different HIV transcripts produced before ART and the rate at which they decay after ART initiation during acute HIV infection. Even during untreated infection, most cells (~90%) have blocks at some stage of transcription. Furthermore, different HIV transcripts decline at different rates on ART, with the fastest decay of cells making completed and intact HIV RNA. Our results suggest that intrinsic or extrinsic immune responses act selectively to either reduce particular stages of HIV transcription or cause selective killing of cells making particular HIV transcripts.

## INTRODUCTION

Despite the potency of antiretroviral therapy (ART) to suppress human immunodeficiency virus (HIV) replication, a reservoir of infectious proviruses persists, even after years of HIV suppression. This reservoir includes latently infected CD4+ T cells, which do not produce virions constitutively but can be induced by activation to produce infectious virus (1–3). Reactivation from a small subset of these cells is thought to be responsible for the plasma virus that rebounds after ART interruption (4, 5). The latent HIV reservoir is seeded extremely early during primary HIV infection (6, 7), yet the frequency of HIV-infected cells is significantly impacted by the timing of ART initiation. Multiple studies have shown that people with HIV (PWH) who were treated during acute infection have lower levels of HIV DNA and RNA (8–12), limited virus diversity (13, 14), lower levels of immune activation (8), and a greater likelihood of becoming post-treatment controllers (15, 16).

Even though ART blocks rounds of new infection, it does not prevent HIV transcription in cells that were already infected, providing a mechanism for the persistence of HIV-transcribing cells (17–19). Those cells that are spontaneously transcribing HIV RNA *in vivo* may lack or may have overcome some barriers to expressing HIV that may not be reversible in other infected cells. Expression of viral products from both intact and defective proviruses may contribute to HIV-associated immune activation and inflammation (20), which are thought to underlie the organ damage and reduced life expectancy that persist despite suppressive ART (21). In addition, intact proviruses that are already transcribing HIV RNA would be poised to initiate rebound when ART is interrupted. In support of this hypothesis, at least four studies have shown that different forms of cell-associated HIV RNA correlate with time to rebound after ART interruption (22–25). Furthermore, one study found that in about half of people who interrupted ART, Pol sequences from the rebound virus matched those from cell-associated HIV RNA prior to ART interruption (26).

Using a panel of assays that can simultaneously quantify multiple different regions of HIV RNA, we have recently shown significant, sequential decreases in the levels of initiated, 5′-elongated, completed, and multiply spliced HIV transcripts in cells from the blood and tissues of ART-suppressed individuals (18, 27, 28). These differences were not explained by differences in assay efficiencies, RNA stabilities, or proviral mutations (18, 27). Using replicate dilutions, we showed that most HIV-infected CD4+ T cells from the blood have initiated HIV transcription, while successively smaller fractions expressed 5′-elongated, polyadenylated (completed), and multiply spliced HIV transcripts, indicating heterogeneous populations of HIV-transcribing cells (18). Ex vivo activation caused successively greater increases in elongated, completed, and multiply spliced transcripts, suggesting a series of reversible blocks to HIV transcriptional elongation, completion, and splicing (18, 29). It is unclear to what degree these blocks to HIV transcription exist prior to the start of ART or whether they change with time on ART.

In addition to differing in their processivity and splicing, HIV transcripts may or may not contain deletions and/or hypermutations, depending on whether they are transcribed from intact or defective proviruses. Using the intact proviral DNA assay (IPDA), which quantifies both defective and “intact” proviral DNAs, studies have shown that cells harboring intact HIV DNA decay faster than defective HIV DNA after years on ART (30–32). Recently, we have developed the novel intact viral RNA assay (IVRA), which uses droplet digital reverse transcriptase PCR (dd-RT-PCR) and the primer probe sets from the IPDA to classify HIV transcripts as defective or likely intact. (33). However, it remains unclear whether HIV transcripts that differ in their processivity or presence/absence of mutations also differ in the degree to which they stimulate intrinsic antiviral defenses or extrinsic immune responses that may help facilitate clearance of HIV-infected cells. A recent study has shown that the proportion of proviruses making 5′-elongated HIV RNA decreases with time on ART (19), suggesting either a selective clearance of infected cells transcribing elongated HIV transcripts or an increase in the block to HIV transcriptional elongation over time. Another study observed a faster decay of multiply spliced HIV RNA compared to unspliced HIV RNA after acute ART treatment (34), suggesting that the selection processes may also depend on the type of HIV transcripts being made.

Further studies are needed to understand how the timing of ART initiation and the duration of ART affect the heterogeneous populations of HIV-transcribing cells, including a detailed characterization of the different HIV transcripts produced before ART and the rate at which different HIV transcripts decay on ART. We hypothesized that more processive (completed and/or spliced) HIV transcripts would decay faster than less processive (short or 5′-elongated) ones and that intact HIV RNA would decay faster than defective HIV RNA. To investigate these questions, we quantified multiple different types of HIV transcripts and the corresponding HIV DNA regions (or proviruses) in longitudinal blood samples obtained before ART initiation (T1) and after 6 months (T2) and 1 year (T3) of suppressive ART in 16 individuals who initiated ART during acute HIV infection.

## RESULTS

### Study participants and study design

The study participants included 16 individuals from the University of California, San Francisco (UCSF), Treat Acute HIV cohort (35), who were diagnosed during acute HIV infection between January 2016 and January 2020 and staged according to the Fiebig classification (Table 1). All participants started ART at the time of diagnosis, at a median of 44 days after the estimated date of detectable infection (36). Eligible participants consented to participate, were provided same-day ART initiation with tenofovir/emtricitabine (TDF/FTC, then TAF/FTC once available in 2016) plus dolutegravir (DTG), and were linked to clinical care (37). At each visit, detailed interviews included questions regarding current medications, medication adherence, intercurrent illnesses, and hospitalizations. In addition, peripheral blood sampling at each visit was performed to measure plasma HIV RNA (Abbott Real Time PCR assay, limit of detection <40 copies/mL). For the current study, blood samples were taken prior to ART initiation (time point 1 or T1) and up to two suppressed time points (T2 and T3), with T2 occurring at a median of 24 weeks (~6 months) after ART initiation and T3 at a median of 62 weeks (~1 year) after ART initiation. All study participants obtained suppressed viremia with <40 copies/mL at T2. The median age of the participants at the time of diagnosis was 28 years, and the median pre-ART CD4+ T-cell count was 514 cells/µL. Additional characteristics of the study population can be found in Table 1.

**TABLE 1:** Demographics and baseline characteristics^*a*^

Characteristic	Value (*n* = 16)
Age at diagnosis (years)	28 (21–45)
Male, *n* (%)	100
Race/ethnicity, *n* (%)	
White	37.5
Asian	18.75
Latino	12.5
African American	6.25
Pacific Islander	6.25
Other	18.8
Time from EDDI^*b*^ to HIV treatment (days)	44 (13–135)
Pre-ART HIV-1 RNA (log10 copies/mL)	5.2 (0.8 to >7)
Pre-ART CD4+ T-cell count (cells/µL)	514 (246–1,131)
Fiebig stage at time of ART initiation, *n* (%)	
Fiebig 1	25
Fiebig 2	12.5
Fiebig 3	6.25
Fiebig 5	56.25

^*a*^ Values are reported as median with range, unless specified otherwise.

^*b*^ EDDI, estimated date of detectable infection.

### Vast reduction in HIV DNA levels during the first 6 months of ART

**Fig 1:**
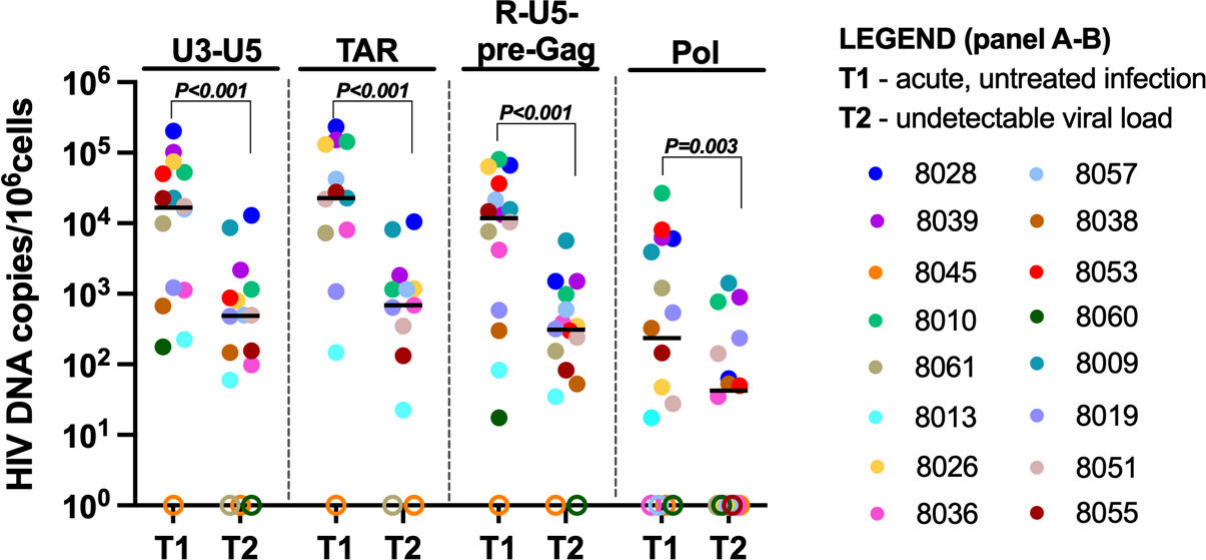
Significant decreases in HIV DNA levels during the first 6 months of ART. Total cellular DNA was extracted and the levels of U3–U5 (RdTh), TAR, R-U5-pre-Gag (LLTR), and Pol HIV DNA were measured by ddPCR before (T1) and after 6 months of suppressive ART (T2). The levels of each HIV DNA region were expressed as the number of copies per 10^6^ cells (assuming 1 µg of total DNA corresponds to 160,000 cells). Horizontal lines indicate medians; different colors indicate individual study participants, and open circles indicate undetectable values. *P* values (two tailed) were calculated using the Wilcoxon matched-pair signed-rank test.

We measured the levels of various HIV DNA regions (corresponding to different HIV RNA transcripts) in blood CD4+ T cells before (T1) and after initiation of ART (T2 and T3), including (i) the U3–U5 long terminal repeat (LTR) (this HIV DNA region contains the sequence targeted in the assay for U3-polyadenylated or “completed” HIV transcripts, and both assays share the same forward primer and probe); (ii) the transactivation response region (TAR, corresponding to initiated HIV RNA); (iii) the R-U5-pre-Gag region (“LLTR,” corresponding to 5′-elongated HIV RNA); and (iv) the Pol region (corresponding to mid-transcribed, unspliced HIV RNA). HIV DNA levels varied greatly between study participants (4–5 log_10_), and we observed one person with no detectable HIV DNA (Fig. 1). The levels of most HIV DNA regions were comparable to each other, except for the Pol region, which was lower than the other three regions even before ART. From T1 to T2, during the first 6 months of ART, levels of all four HIV DNA regions declined significantly (all *P* ≤ 0.003, Fig. 1). However, no significant reductions were observed between 6 months (T2) and 1 year (T3) on ART (Fig. S1). Although this cohort consisted of individuals who were all treated during acute infection, we observed large interperson variability in HIV levels. Consequently, we conducted a more detailed analysis by categorizing all participants based on their Fiebig stage at T1. Due to the low numbers of individuals who initiated ART during each of Fiebig stages I–III and the absence of individuals from stage IV, we grouped people from Fiebig I–III and compared them to Fiebig V. Before ART (T1), no differences in HIV DNA levels were observed between Fiebig groups I–III and V (Fig. S2A), and the reduction in the different HIV DNA regions from T1 to T2 was comparable between the two Fiebig groups (Fig. S2B).

### Differences between levels of various HIV transcripts before ART

**Fig 2:**
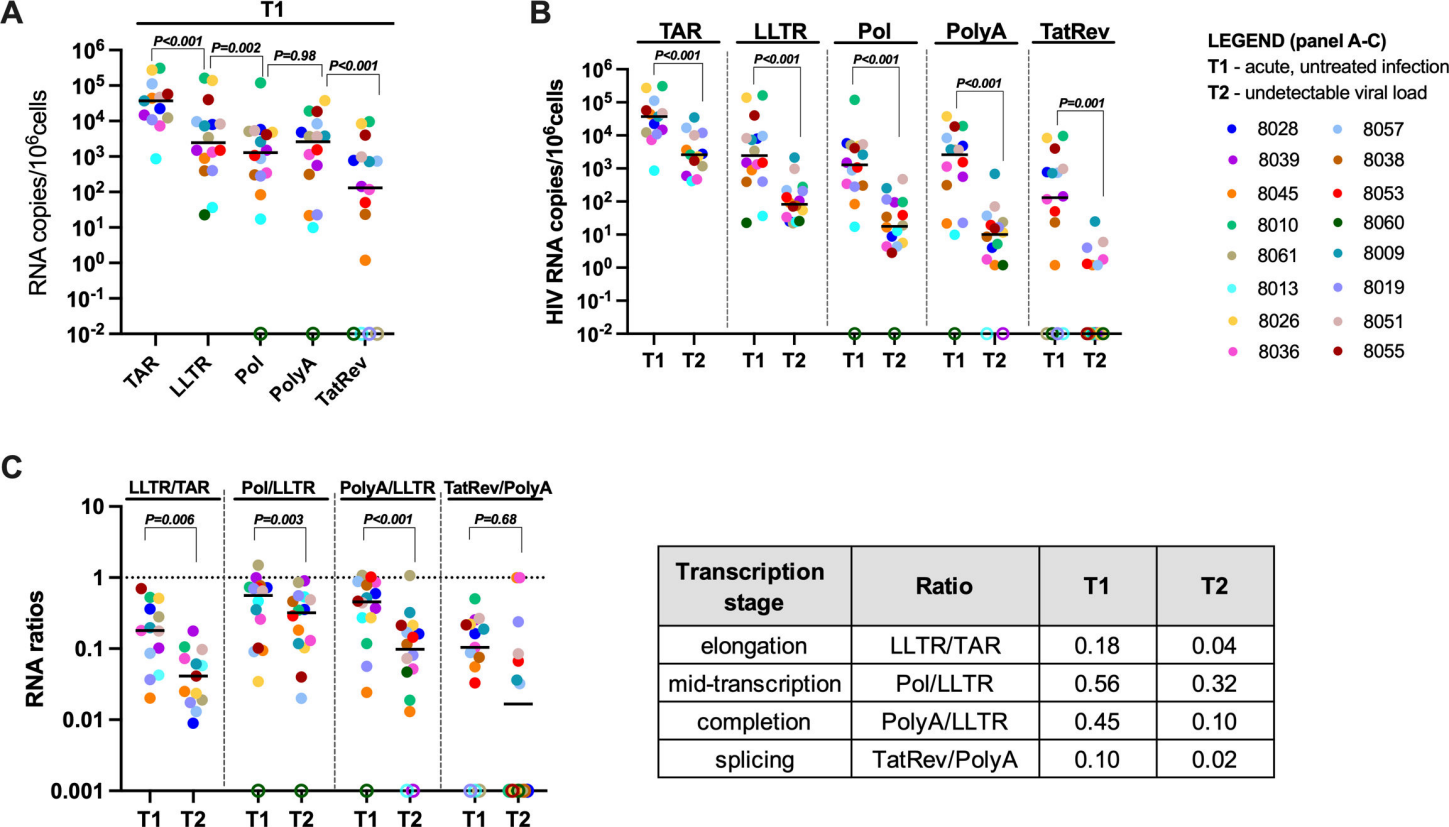
Progressive reductions in HIV transcription after initiation of ART. Total cell-associated RNA was extracted, and the progression through different stages of HIV transcription was quantified by RT-ddPCR. The levels of initiated (TAR), 5′-elongated (LLTR), mid-transcribed/unspliced (Pol), completed (PolyA), and multiply spliced (TatRev) HIV transcripts were measured and expressed as the number of copies per 10^6^ cells (assuming 1 µg of total RNA corresponds to 10^6^ cells). (**A**) HIV RNA transcripts before suppressive ART (T1) and (**B**) before (T1) and after 6 months (T2) of ART. (**C**) The ratios of one HIV RNA to another to evaluate the progression through HIV transcriptional elongation, mid-transcription, completion, and splicing before (T1) and after 6 months of suppressive ART (T2). Ratios are independent of effects at prior stages of HIV transcription and independent of infection frequency or normalization to cell numbers. Shown are the proportion of (i) all HIV transcripts that were elongated (LLTR/TAR), (ii) elongated HIV transcripts that were mid-transcribed (Pol/LLTR) or (iii) completed (PolyA/LLTR), and (iv) completed transcripts that were multiply spliced (TatRev/PolyA). (**A–C**) Horizontal lines indicate medians; different colors indicate individual study participants; and open circles indicate undetectable values. *P* values (two tailed) were calculated using the Wilcoxon signed-rank test.

Levels of initiated (TAR), 5′-elongated (LLTR), mid-transcribed/unspliced (Pol), completed (PolyA), and multiply spliced (TatRev) HIV transcripts were quantified by RT-ddPCR at times T1–T3 and normalized to total cellular transcription (1 µg of RNA, corresponding to ~10^6^ CD4+ T cells). At T1, during untreated acute infection, levels of initiated HIV transcripts were significantly higher than 5′-elongated HIV transcripts (*P* < 0.001, Fig. 2A), and levels of completed HIV transcripts were significantly higher than levels of multiply spliced TatRev transcripts (*P* < 0.001). Transcripts that are 5′ elongated were slightly more abundant than mid-transcribed (Pol) transcripts (*P* = 0.002), but we did not find significant differences between 5′-elongated and completed HIV transcripts, or between mid-transcribed and completed HIV transcripts (Fig. 2A).

**Fig 3:**
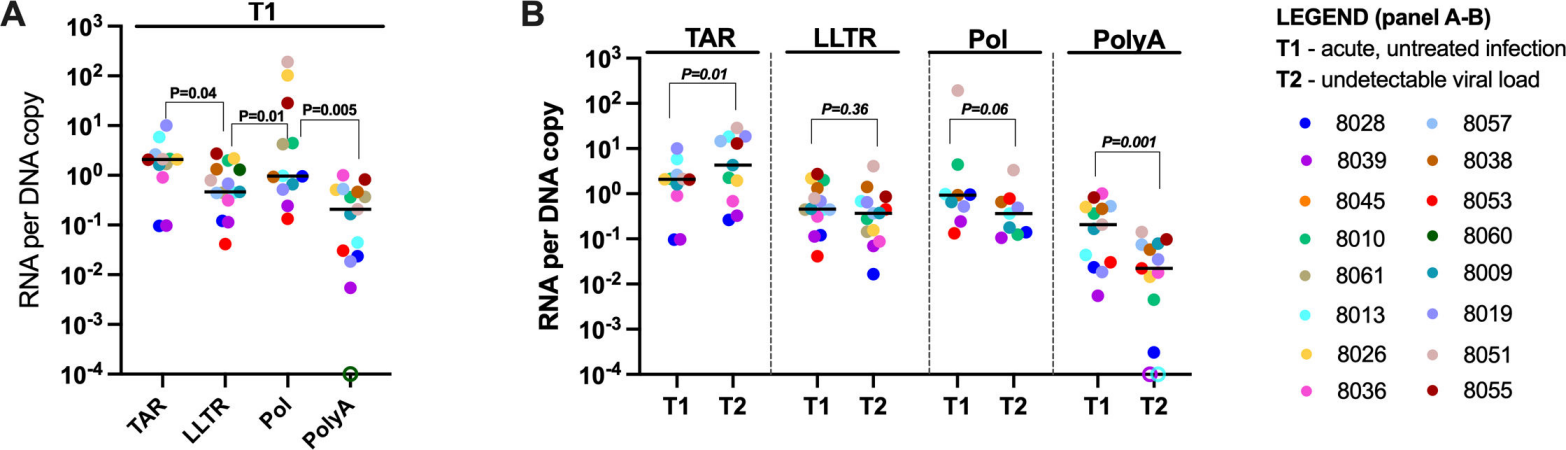
Completed (PolyA) transcripts decayed faster after initiation of ART, even after adjusting for differences in HIV DNA levels. The levels of each HIV transcript were normalized to the corresponding HIV DNA to account for differences in infection frequency and proviral mutations. The HIV transcripts per DNA copy are shown (**A**) before (T1) and (**B**) before (T1) and after 6 months of suppressive ART (T2). (**A and B**) Horizontal lines indicate medians; different colors indicate individual study participants; and open circles indicate undetectable values. *P* values (two tailed) were calculated using the Wilcoxon signed-rank test.

Since levels of various HIV transcripts can also be affected by the relative frequencies of mutations at each HIV DNA region, we also normalized each HIV transcript to levels of the corresponding HIV DNA region (both normalized to 10^6^ cells using nucleic acid mass) to express the HIV RNA/DNA (average transcription per provirus). HIV DNA was measured by the same assay used for the RNA (Fig. 1), except for completed HIV transcripts (which were normalized to the U3–U5 DNA) and multiply spliced HIV transcripts (for which there is no corresponding HIV DNA). After normalization to HIV DNA, we still saw that initiated HIV RNA/DNA was greater than 5′-elongated HIV RNA/DNA (*P* = 0.04, Fig. 3A), suggesting a block to elongation. In contrast, 5′-elongated HIV RNA/DNA was not greater than mid-transcribed HIV RNA/DNA, but levels of mid-transcribed HIV RNA/DNA were significantly higher than levels of completed HIV RNA/DNA (*P* = 0.005, Fig. 3A).

Initiated (TAR) and multiply spliced (TatRev) HIV RNA levels tended to be lower in individuals in Fiebig I–III than Fiebig V (*P* = 0.05 and *P* = 0.08, respectively; Fig. S3A) at T1, but this difference did not persist when HIV RNA levels were normalized to the corresponding HIV DNA region (Fig. S3B).

### Progressive reductions in HIV transcription after initiation of ART

All HIV RNA levels decreased significantly from T1 (pre-ART) to T2 (6 months, all *P* ≤ 0.001; Fig. 2B). Moreover, we observed progressively greater reductions from T1 to T2 in initiated (median fold decline of 10), 5′-elongated (13-fold), mid-transcribed (28-fold), completed (227-fold), and multiply spliced HIV transcripts (1,236-fold). To determine whether the differences between the decay rates of various HIV transcripts were statistically significant, we calculated the decay of each transcript by dividing its value at T1 by that at T2 and then compared this ratio with the decay rates of the other transcripts. After correcting for multiple testing, we found that completed HIV transcripts (PolyA) decayed faster than initiated (TAR, *P* < 0.001) and 5′-elongated (LLTR, *P* = 0.007) transcripts (Table S1). In the 11 individuals from whom samples were available at T3 (~1 year), we did not observe significant decreases in any HIV transcript from T2 to T3 (Fig. S4A).

When comparing Fiebig groups, we did not detect significant differences in HIV RNA levels between Fiebig groups I–III and V at T2 (Fig. S5). HIV transcripts that are 5′ elongated, mid-transcribed, and completed declined significantly from T1 to T2 in both groups (Fig. S5). Significant decreases in initiated and multiply spliced TatRev HIV RNA were observed only in individuals treated during Fiebig V, but the power to detect changes was greater in this group due to the larger number of individuals and greater number with detectable, multiply spliced HIV RNA at T1.

To evaluate the progression through blocks to HIV transcriptional elongation, completion, and splicing in a manner that is independent of infection frequency or progression through prior blocks, we also calculated the ratio of one HIV RNA to another at a given time point and compared between time points. We compared the proportions of (i) initiated HIV transcripts that were 5′ elongated (LLTR/TAR), (ii) 5′-elongated HIV transcripts that were mid-transcribed (Pol/LLTR) or (iii) completed (PolyA/LLTR), and (iv) completed transcripts that were multiply spliced (TatRev/PolyA). From T1 to T2, there was a significant reduction in HIV 5′ elongation, mid-transcription, and completion (all *P* ≤ 0.006, Fig. 2C), suggesting immune selection and/or progressive HIV transcriptional silencing. From T2 to T3, the median values of each ratio decreased further, but we only detected a significant decrease in Pol/LLTR (*P* = 0.04, Fig. S4B).

### Completed transcripts decayed faster after initiation of ART, even after adjusting for the changes in HIV DNA

To account for differences in how the relative frequencies of various proviral regions or mutations at these regions may change over time, we also normalized each HIV transcript to levels of the corresponding HIV DNA region at the same time point and then compared the HIV RNA/DNA across time points. From T1 to T2, we observed a significant decrease in completed HIV RNA per provirus (*P* = 0.001) and a trend toward reduction in mid-transcribed (Pol) HIV RNA per provirus (*P* = 0.06, Fig. 3B). However, we did not detect reductions in initiated HIV RNA/DNA or 5′-elongated HIV RNA/DNA, suggesting that the reductions in these HIV transcripts may have been driven by changes in the corresponding HIV DNA regions (Fig. 3B). From T2 to T3, we did not observe any further decrease in any type of HIV RNA per provirus (Fig. S6).

The decrease in completed (PolyA) HIV RNA per provirus from T1 to T2 was significant only for individuals treated during Fiebig stage V (Fig. S7). However, the power to detect a decrease was lower in the Fiebig I–III group due to the lower number of individuals and greater number with 0 or undefined values.

### Intact HIV RNA, but not intact DNA, decreased faster than defective sequences

**Fig 4:**
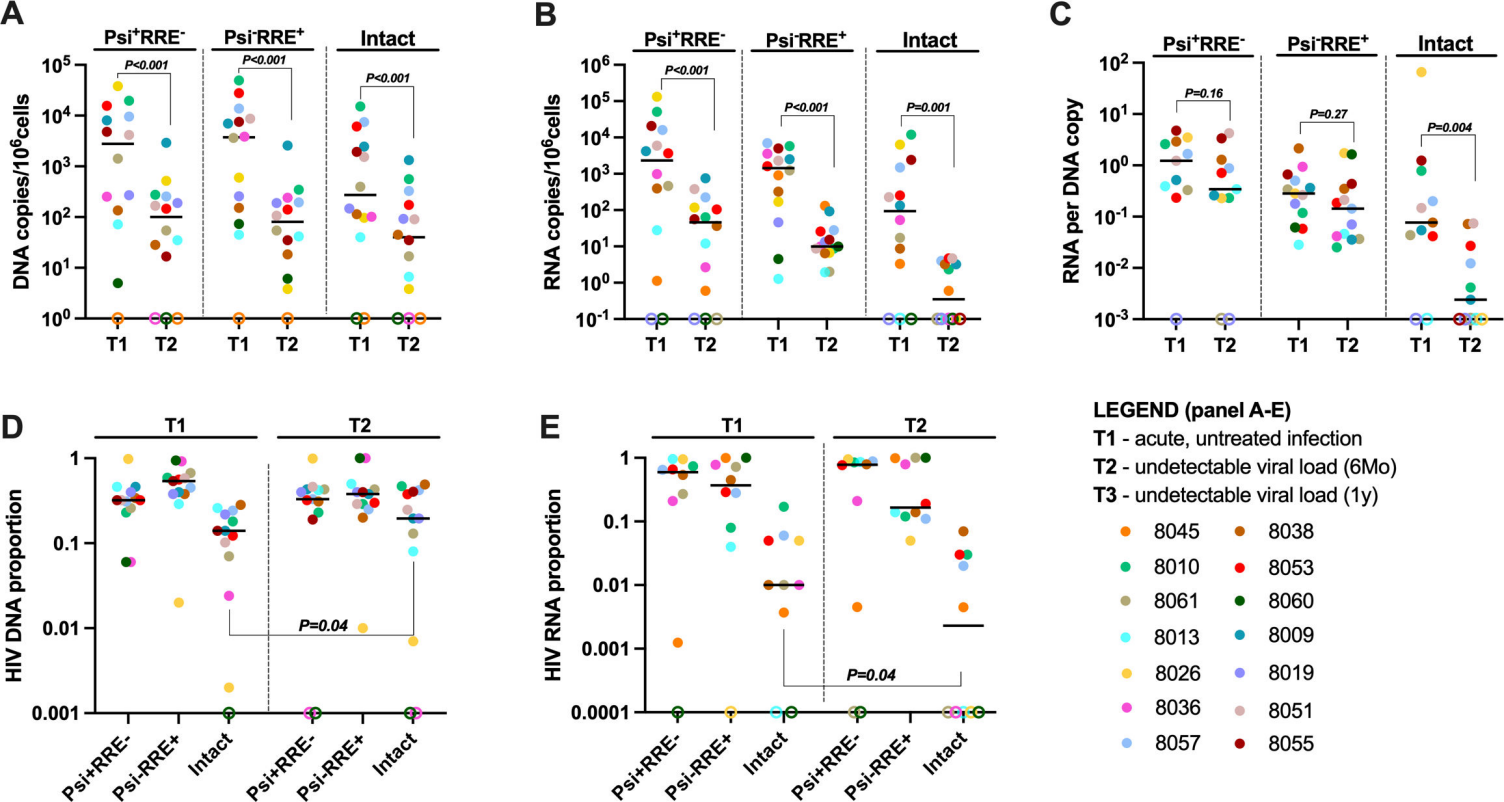
Intact HIV RNA, but not intact DNA, decreased faster than defective sequences. (**A**) Total cellular DNA was extracted, and the levels of 3′-defective (Psi+RRE−), 5′-defective (Psi−RRE+), and intact (Psi+RRE+) HIV DNAs were measured using the intact proviral DNA assay before (T1) and after 6 months of suppressive ART (T2). The levels of each HIV DNA region were expressed as the number of copies per 10^6^ cells (assuming 1 µg of total DNA corresponds to 160,000 cells). (**B**) Total cell-associated RNA was extracted, and the levels of 3′-defective (Psi+RRE−), 5′-defective (Psi−RRE+), and intact (Psi+RRE+) HIV RNAs were measured using the intact viral RNA assay before (T1) and after 6 months of suppressive ART (T2). The levels of each HIV transcript were expressed as the number of copies per 10^6^ cells (assuming 1 µg of total RNA corresponds to 10^6^ cells). (**C**) The levels of each HIV transcript (3′ defective, 5′ defective, and intact) were normalized to the corresponding HIV DNA to account for differences in infection frequency and proviral mutations. HIV transcripts per provirus are shown before (T1) and after 6 months of suppressive ART (T2). (**D and E**) The proportions are shown before (T1) and after 6 months (T2) of suppressive ART of (**D**) 3′-defective, 5′-defective, and intact HIV DNAs, and (**E**) 3′-defective, 5′-defective, and intact HIV RNAs. (**A–E**) Horizontal lines indicate medians; different colors indicate individual study participants; and open circles indicate undetectable values. *P* values (two tailed) were calculated using the Wilcoxon signed-rank test.

Intact (packaging signal [Psi]+rev response element [RRE]+) and defective (Psi−RRE+ and Psi+RRE−) HIV DNAs were measured by ddPCR, and intact and defective HIV RNAs were measured by dd-RT-PCR, as previously described (33). From T1 to T2, 3′-defective (Psi+RRE−), 5′-defective (Psi−RRE+), and intact (Psi+RRE+) HIV DNAs decreased significantly (Fig. 4A, all *P* < 0.001), with median fold declines of 38, 67, and 23, respectively. However, we did not observe statistically significant differences between the decay (T1/T2) of intact and defective HIV DNAs. From T2 to T3, we observed small but significant reductions in 3′-defective (median fold decline of 2.1, *P* = 0.008) and intact (median fold decline of 5.0, *P* = 0.02; Fig. S8A) HIV DNAs. From T1 to T2, the reduction in intact and defective HIV DNAs did not differ significantly between individuals treated during Fiebig groups I–III and V (Fig. S9).

From T1 to T2, we also observed significant decreases in intact HIV RNA and both types of defective HIV RNA (Fig. 4B, all *P* ≤ 0.001). In contrast to HIV DNA, the median fold decline (T1/T2) in intact HIV RNA (372-fold) was approximately 7.9-fold greater than 3′-defective RNA (47-fold, *P* = 0.06) and 6.6-fold greater than 5′-defective RNA (56-fold). The difference between the decay rate (T1/T2) of intact and 3′-defective HIV RNAs approached statistical significance (*P* = 0.06), while the difference between decay of intact and 5′-defective HIV RNAs did not. Consistent with our previous cross-sectional study in ART-suppressed patients (33), 3′-defective HIV RNA was most abundant at T2 (median 46 copies/10^6^ cells), followed by 5′-defective (10 copies/10^6^ cells) and intact (0.35 copies/10^6^ cells) HIV RNAs.

The decreases in defective and intact HIV RNAs from T1 to T2 were significant only for those treated during Fiebig stage V (*P* = 0.008 for all, Fig. S10A). However, we had lower power to detect changes in the Fiebig I–III group because of the smaller number of individuals and the greater numbers with undetectable values at T1. By T2, intact HIV RNA levels were significantly lower in those treated during stage I-III than stage V (*P* = 0.047; Fig. S10B).

Next, we calculated the ratios of each HIV RNA type to the corresponding HIV DNA in order to express the average levels of each HIV RNA per provirus. At T1, the median ratio of intact RNA to intact HIV DNA was ~0.1, suggesting that at most 10% of intact proviruses are making intact HIV RNA. From T1 to T2, we observed a significant reduction in intact RNA per intact provirus, but not in either type of defective RNA per provirus (Fig. 4C, *P* = 0.004). The median fold decline (T1/T2) in intact HIV RNA per intact HIV DNA (23-fold) was approximately 10-fold greater than the decline in 3′-defective (2.0-fold) or 5′-defective (2.5-fold) HIV RNA per provirus, and intact HIV RNA/DNA tended to decay faster than either type of defective HIV RNA/DNA (both *P* = 0.07). From T2 to T3, we did not detect significant changes in any type of HIV RNA per provirus (Fig. S8C).

The decrease in intact HIV RNA per intact provirus was significant only for individuals treated during Fiebig stage V (*P* = 0.03, Fig. S11A), but we had less power to detect a decrease in the Fiebig I–III group, which had fewer individuals and more with 0 or undefined values. By T2, levels of intact HIV RNA per intact provirus were lower in individuals treated during Fiebig stages I–III (*P* = 0.02, Fig. S11B).

Congruent with some (30) but not all (38) studies, the median ratio of intact to total HIV DNA was approximately 10% at T1, before the start of ART (Fig. 4D). In contrast, only about 1% of the HIV RNA was intact at T1. During the first 6 months of ART, the proportion of intact HIV DNA increased slightly (*P* = 0.04, Fig. 4D), while the proportion of intact HIV RNA decreased (*P* = 0.04, Fig. 4E)

### HIV DNA and RNA levels at T1–T3 correlate inversely with baseline CD4+ T-cell count

**Fig 5:**
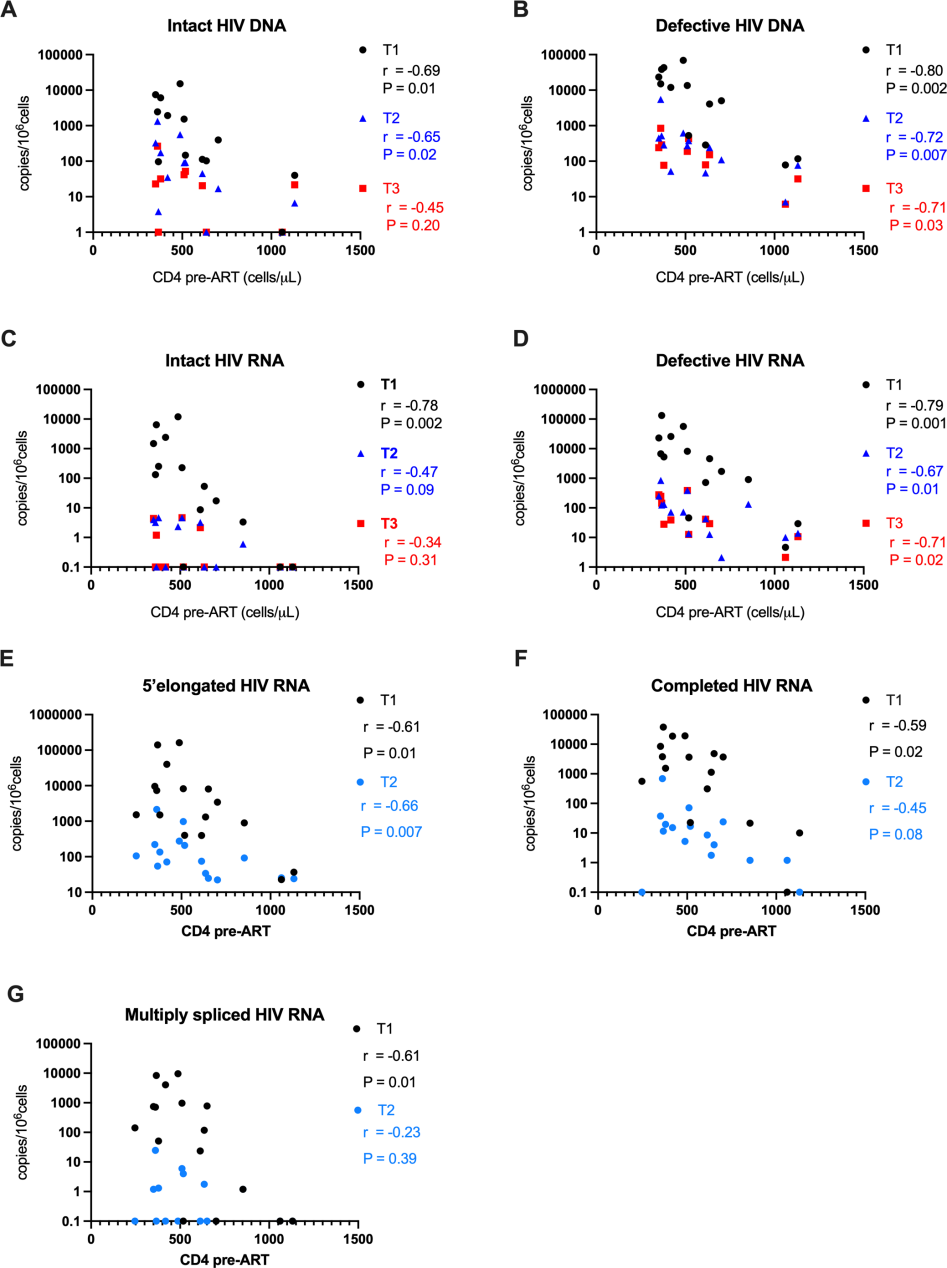
Correlations between pre-ART CD4+ T cells (T1) and HIV DNA and RNA measures at T1, T2, and T3. HIV DNA (**A and B**) and HIV RNA (**C–G**) levels (*y*-axis) at T1 (black symbols), T2 (blue), and T3 (red) were correlated with the absolute CD4 count at T1 (*x-*axis) using Spearman correlations. Spearman *r* values and *P* values (two tailed) are listed for each correlation.

The CD4 count at T1 correlated inversely with various HIV DNA and RNA levels at T1–T3, including (i) intact HIV DNA (*r* = −0.69, Fig. 5A), defective HIV DNA (*r* = −0.80, Fig. 5B), intact HIV RNA (*r* = −0.78, Fig. 5C), defective HIV RNA (*r* = −0.79, Fig. 5D), 5′-elongated HIV RNA (*r* = −0.61, Fig. 5E), completed HIV RNA (*r* = −0.59, Fig. 5F), and multiply spliced HIV RNA (*r* = −0.61, Fig. 5G) at T1 (all *P* < 0.05); (ii) intact HIV DNA (*r* = −0.65, Fig. 5A), defective HIV DNA (*r* = −0.72, Fig. 5B), defective HIV RNA (*r* = −0.67, Fig. 5D), and 5′-elongated HIV RNA (*r* = −0.66, Fig. 5E) at T2 (all *P* < 0.05); and (iii) defective HIV DNA and RNA (both *r* = −.071, *P* < 0.05; Fig. 5B and D) but not intact HIV DNA or RNA at T3.

### The plasma viral load at T1 correlates with most HIV RNAs and DNAs at T1 but not at T2 or T3

During untreated infection (T1), we observed strong correlations between the plasma viral load and most HIV RNAs and DNAs (all *P* ≤ 0.004, except Pol HIV DNA and intact HIV DNA; Fig. S12A) but not HIV RNA:DNA ratios. Most HIV RNAs and DNAs also correlated with each other.

In contrast, none of the HIV RNAs or DNAs at T2 or T3 correlated significantly with the viral load at T1. At T2, initiated (TAR) and 5′-elongated (LLTR) HIV transcripts correlated with most other HIV transcripts and with intact and defective HIV DNAs (Fig. S12B). U3–U5 HIV DNA correlated with all the other HIV DNA levels, and intact HIV DNA also correlated with defective and Pol HIV DNAs at T2. Intact HIV RNA correlated with defective HIV RNA, intact HIV DNA, and Pol RNA/DNA. Most HIV RNA/DNA levels correlated with each other, while we observed few correlations with HIV RNA (except Pol HIV RNA/DNA and intact HIV RNA) or HIV DNA.

At T3, we observed correlations between initiated (TAR), 5′-elongated (LLTR), Pol, and completed (PolyA) HIV RNAs, and between these HIV RNAs and intact HIV DNA (Fig. S12C). Intact HIV DNA also correlated with TAR RNA/DNA and LLTR RNA/DNA. Intact HIV RNA correlated with defective RNA but not other HIV RNA or DNA levels. Of the different types of HIV DNA, intact HIV DNA showed the greatest number of correlations with different HIV transcripts during both suppressed time points (Fig. S12B and C).

## DISCUSSION

Early ART is associated with preserved immune functions, decreased viral escape from host immunity, and smaller reservoir sizes (8–12). A recent study demonstrated that the decay of intact and defective HIV DNAs after ART initiation during acute infection exhibits a biphasic decay pattern (35), consistent with other studies using different HIV DNA assays (9, 10, 39, 40). This pattern is similar to that in individuals initiating ART during chronic infection (10, 31), except that the second phase of intact HIV DNA decay (weeks 5–26) is much faster than previously described in chronic treated PWH (38) but very consistent with decay rates in other studies in acute-treated PWH using different HIV DNA assays (e.g., intact, total, or integrated HIV DNA) (10). The earlier that ART is initiated, the faster and more pronounced the decay during the initial phase (9, 10, 12, 34, 41), in particular, that of productively infected cells (34, 39, 41), suggesting that early ART leads to accelerated decay of HIV-transcribing cells. The current study aimed to build on previous research by investigating HIV transcription during untreated acute infection and measuring how different types of HIV transcripts decay over time after initiation of ART during acute infection.

No prior studies have investigated whether or to what degree the blocks to HIV transcriptional elongation, completion, and splicing that likely underlie latent infection are present before the start of ART (18, 27, 42). This time period before ART initiation may be important for the establishment of latent infection, since the unique viruses that grew out from viral outgrowth assays on ART were most similar to plasma HIV RNA sequences from just prior to ART initiation (43). Although one might predict that the pattern of HIV transcripts during untreated acute infection is dominated by productively infected cells making large quantities of full-length genomic HIV RNA, we observed significant differences between the levels of initiated and 5′-elongated HIV RNA transcripts, and between completed and multiply spliced HIV transcripts, at T1. Although we were not able to normalize the multiply spliced HIV RNA to DNA, the difference between initiated and 5′-elongated HIV transcripts persisted even after normalization to the corresponding HIV DNA regions, suggesting that it is not explained by differences in the frequency of proviral mutations. These findings suggest that even during untreated acute infection, some infected cells have blocks to HIV transcriptional elongation and multiple splicing (respectively). In addition, there may be preferential immune selection against cells in which HIV transcription has proceeded through these stages. We also observed a difference between levels of mid-transcribed and completed HIV RNA per provirus, which could indicate a block to distal transcription or immune selection against cells making completed HIV transcripts.

Our data suggest that prior to ART initiation, some infected cells may have blocks to various stages of HIV transcriptional processivity, which could prevent virus production and impact immune responses. Several prior studies in untreated individuals have suggested that the majority of HIV-infected cells are not productively infected. One study of ART-naïve individuals showed that ex vivo T-cell activation dramatically increased the frequency of peripheral blood cells in which unspliced HIV RNA could be detected by *in situ* hybridization, while another study showed that circulating cells with intracellular unspliced HIV RNA were at least 30 times more abundant than those with multiply spliced or virion-attached viral RNA (44). These findings could be explained if many of the HIV-infected cells have reversible blocks to HIV transcriptional processivity and/or splicing that inhibit or prevent virus expression even prior to the initiation of ART.

Given the high degree of ongoing replication that exists in untreated infection and the fact that a median of ~10% of the HIV DNA was intact, we were also surprised to find that such a small percentage of all the cell-associated HIV RNA was intact (median of ~1%). In addition, the median ratio of intact HIV RNA to intact HIV DNA at T1 was around 0.1, suggesting that at most 10% of intact proviruses are transcribing intact HIV RNA during untreated infection and that the remainder (~90%) have blocks at some stage of transcription. Both the 5′-defective (Psi−RRE+) and 3′-defective (Psi+RRE−) HIV RNAs could result from deletions or hypermutations in the Psi or RRE regions, respectively, of the proviruses from which they were transcribed. The 3′-defective HIV RNA could also result from premature termination of 5′-initiated HIV transcription between the Psi and RRE regions, including HIV RNA from intact proviruses. Premature termination would not be expected to generate 5′-defective HIV RNA unless the transcription originated from the 3′ LTR, but 5′-defective HIV RNA could also be singly spliced HIV RNA (including from intact proviruses). However, even if some of the defective (single-positive) HIV RNA is from intact proviruses, the ratios of 3′-defective and 5′-defective HIV RNAs to DNA provide an upper limit on the proportion of these defective proviruses that are transcribing elongated HIV RNA. Since spliced HIV RNA is generally less abundant than unspliced HIV RNA, our results also suggest that the majority of all HIV RNA in untreated infections is transcribed from defective proviruses and/or prematurely terminated.

After 6 months of ART, we observed large decreases in all HIV DNA regions and all HIV transcripts. However, we detected progressively greater reductions in initiated, 5′-elongated, mid-transcribed, completed, and multiply spliced HIV transcripts, and completed HIV RNA decayed faster than initiated or 5′-elongated HIV RNA. By measuring the ratios of different HIV RNA types, we observed significant reductions in HIV 5′ elongation, mid-transcription, and completion, with the most significant reduction occurring at the stage of completion. After normalizing each HIV RNA to the corresponding HIV DNA, we found a significant decay in completed HIV transcripts per provirus and a trend toward a decrease in mid-transcribed (Pol) HIV RNA per provirus.

We hypothesize that the clearance of productively infected cells contributes to the fast decay in multiply spliced and completed HIV RNA. Plasma HIV RNA decays rapidly after starting ART, with a first-phase decay that has been attributed to clearance of productively infected CD4+ T cells; a slower, second-phase decay attributed to clearance of a longer lived, productively infected cell type; and a third-phase decay attributed to the clearance of latently infected cells (45, 46). Multiply spliced TatRev HIV RNA serves as a template to translate HIV Tat and Rev proteins, which are necessary for productive infection, and multiply spliced HIV RNA has been reported to be a surrogate marker for productive infection (22, 34). Prior studies have shown that multiply spliced HIV RNA declines faster than unspliced HIV, which has been attributed to the rapid clearance of productively infected cells (34, 47–49). In addition, productive infection requires unspliced HIV RNA that is processive enough to include both the Psi (for packaging into virions) and the RRE (for Rev-assisted nuclear export).

While we acknowledge that the clearance of productively infected cells may contribute to the rapid decline in completed and multiply spliced HIV RNA, this explanation does not seem sufficient to explain all of the differences we observed between the decay of various HIV transcripts. First, non-productively infected cells are much more abundant than productively infected cells even in untreated infection (22, 34), and the decrease in HIV DNA from T1 to T2 seems too large to be explained solely by clearance of productively infected cells. Second, the vast majority of CD4+ T cells with intact proviruses are distinct from the rapidly decaying, productively infected cells (38). Third, if the unspliced HIV RNA in productively infected cells is predominantly full-length genomic HIV RNA (containing one each of the R-U5-pre-Gag, Pol, and U3-PolyA regions), one might expect that clearance of these cells would cause comparable decreases in 5′-elongated, mid-transcribed (Pol), and completed transcripts rather than progressively greater decreases in these HIV transcripts. Moreover, since TAR HIV RNA is present at two copies per full-length HIV RNA, one might expect that TAR HIV RNA would decline faster than the other transcripts, while we observed the opposite.

Instead, our data suggest that the changes on ART are the consequence of immune selective forces that exert differential effects on productively infected cells as well as a spectrum of non-productively infected cells with various degrees of progression through blocks to HIV transcriptional elongation, completion, and splicing. ART blocks new infection in the blood, but the antiretrovirals (ARVs) used in this study should not directly affect HIV transcription, suggesting that the changes we observed on ART are the result of intrinsic or extrinsic immune responses that act selectively to either reduce particular stages of HIV transcription and/or to cause selective killing of cells making particular HIV transcripts. CD8+ T cells have been shown to exert one or more non-cytolytic activities that reduce HIV transcription (50, 51), including the secretion of CAF factor, but it is not clear what stages or blocks to HIV transcription are affected. Longer, more processive HIV transcripts may be more likely to trigger intracellular defenses. For example, full-length HIV RNA has been reported to be a ligand for RIG-I (52, 53). In addition, longer HIV transcripts may contain more protein-coding regions, which have the potential to be translated into HIV proteins. Moreover, the presence of a PolyA tail (in completed HIV transcripts) should facilitate nuclear export, RNA stability, and translation into proteins. Some HIV proteins (protease, Vpr, and Env) can trigger apoptosis (54), and HIV proteins can also be processed and presented on the cell surface, which can trigger killing by extrinsic immune cells. Any of these explanations could explain why cells making longer or more processive HIV transcripts may be cleared at faster rates.

We also found differences between the decay of intact and defective HIV RNA, and between the decay of intact HIV RNA and intact proviruses. Recently, Barbehenn et al. (35) conducted a comprehensive mathematical modeling of the HIV DNA reservoir decay in individuals from the same cohort of acute-treated individuals that were studied here. They observed a biphasic decay of HIV DNA, with an initial rapid decline until approximately 5 weeks of ART, followed by a slower decline up to 1 year of ART. Interestingly, the decay of defective HIV DNA was slightly faster than that of intact HIV DNA during the first phase (5 weeks), in contrast with earlier studies investigating reservoir decay after long-term periods of ART (30, 31, 55). In the current study, we observed no statistically significant difference between the decay of intact and defective HIV DNA from pre-ART (T1) up to 6 months (T2) or 1 year on ART (T3). The accelerated decline of intact HIV DNA over extended periods of time may result from immune mechanisms that differ from those occurring during the first 6 months of ART.

No prior studies have compared the decay of intact and defective HIV RNA. We applied the IVRA (33) to measure the kinetics of intact and defective HIV RNA after starting ART. While we did not detect differences in the decline of intact and defective HIV DNAs, the median fold decline in intact HIV RNA over the first 6 months of ART was approximately seven- to eightfold greater than either type of defective HIV RNA, and this trend persisted even after normalization to the corresponding HIV DNA. These findings suggest that either there is a greater reduction in HIV transcription from intact proviruses and/or that intact HIV RNA may be more likely than defective HIV RNA to trigger immune-mediated processes that mediate clearance of HIV-infected cells. Some of the defective HIV RNA (particularly Psi+RRE−) may be prematurely terminated, but HIV RNA with deletions or hypermutations may also prevent translation of certain HIV proteins. Both processes could impair extrinsic immune responses that require recognition of HIV peptides. By T2, the median ratio of intact HIV RNA to intact HIV DNA had decreased from ~0.1 to ~0.01, suggesting that ~99% of intact proviruses are no longer making intact HIV RNA. Between T2 and T3, we observed a small decrease in intact HIV DNA but no further decrease in intact HIV RNA. The inability to measure further decreases in intact HIV RNA may simply reflect the lower abundance of intact HIV RNA relative to intact DNA, but it is also possible that cells with intact HIV DNA continued to be cleared through different selection processes that occur over longer time spans. At both ART-suppressed time points (T2 and T3), among the different types of HIV DNA, intact HIV DNA correlated strongest with the various HIV transcripts (Fig. S12B and C). This finding suggests that during suppressed HIV infection, at least some of the HIV RNA is transcribed from intact HIV DNA. Notably, this correlation was observed despite the fact that intact HIV DNA levels were lower than defective HIV DNA or HIV DNA assays targeting the LTR, which serves as the promoter for HIV transcription.

It is not clear what immune mechanisms are responsible for the decay in different HIV transcripts and proviruses. Ongoing studies are measuring plasma cytokines (56) and immune responses from the same study participants and time points at which we quantified the HIV RNA and DNA levels, and future studies will attempt to correlate these measures of immune responses with the declines in HIV RNA and DNA. Recent studies have associated HIV-specific CD4+ T cells (57) and interleukin-10 levels (56) with the decay of intact but not defective HIV DNA. Considering that intact HIV RNA serves as a closer proxy to replication competent provirus and is likely more immunogenic than intact HIV DNA, we anticipate that the strong decay in intact HIV RNA observed in the current study is the result of specific immune responses against cells transcribing intact HIV RNA. Nonetheless, the recent Netherlands Cohort Study on Acute HIV infection (NOVA) (57) found no correlation between HIV-specific CD4+ T cells and unspliced HIV RNA, suggesting that mid-transcribed (unspliced) HIV RNA may not be that immunogenic for T-cell responses. Our data may provide an explanation, as we observed a faster decay of completed (polyadenylated), multiply spliced, and intact HIV RNA, which are more likely to be translated into HIV proteins, processed into peptides, and presented to T cells. Together, our data indicate that immune responses against HIV may be dependent on the processivity of the HIV RNA and the presence/absence of mutations.

We also observed significant inverse correlations between CD4+ T-cell count during untreated acute infection and various HIV DNA and RNA levels from the same time point (T1) and 6–12 months post-ART (T2-T3). Although correlations cannot be used to infer causality, it seems logical that more HIV infection (DNA) and transcription (RNA) during untreated infection could lead to greater CD4+ T-cell depletion (lower CD4 count) and/or that declining immune function (lower CD4) could allow for more HIV infection and transcription. The extent of immune damage (CD4 depletion) during untreated infection could also affect residual HIV infection and transcription at later times on ART, although it is also possible that this correlation is driven by an association between the HIV levels at T2 and T3 and those at T1. To our surprise, the inverse correlation was stronger for defective than intact HIV at T2 and was only observed for defective HIV DNA and RNA at T3. However, intact HIV levels were very low and sometimes not detected at T2 and T3, which may have made it more difficult to detect a correlation.

Although the Treat Acute cohort consists of individuals who were all treated during acute infection, we observed large interperson variability in HIV DNA and RNA levels. This variability could be attributed to host and/or viral factors, including host genomic factors (58, 59) and viral sequence diversity (60, 61). Deletions or hypermutations in some HIV DNA regions—such as the LTR, splice donor/acceptor sites, Tat, or Rev—could reduce or eliminate HIV transcription, splicing, or nuclear export. The intactness of the viral genome may dictate HIV transcription, particularly the integrity of the LTR region (which contains the promoter and regulates HIV transcriptional initiation) and the viral transcription factor Tat (which promotes elongation). We have quantified overlapping regions of the HIV LTR region using ddPCR primers and probes that target most of the LTR (U3–U5), the transcription start site (TAR), or the 5′ LTR (R-U5-pre-Gag region). Interestingly, levels of these LTR DNA regions correlated strongly with most HIV transcripts at T1 (all *P* < 0.001, Fig. S12A) but not at T2 or T3, while intact HIV DNA correlated with various HIV transcripts at T2 and T3.

Other limitations of our study should also be acknowledged. One limitation of our study is the infrequency of our sampling compared to the study by Barbehenn et al. (35), which limits our ability to quantify the decay of different HIV transcripts at various times during the first 6 months of ART. Second, most of the acute-treated individuals in this study had not been on ART for a long time, so we did not have later time points and were not able to determine whether different HIV transcripts continue to decline over much longer periods of ART. Third, some of these acute-treated individuals had very low infection frequencies, which may have limited our ability to detect certain HIV DNA regions (Pol), intact proviruses, and some HIV RNA species (e.g., multiply spliced and intact HIV RNA). The lower levels and detection frequency of HIV Pol DNA could be the result of the Pol sequences being less conserved or more mutated compared to the HIV LTR regions. The failure to detect multiply spliced HIV RNA, especially at suppressed time points, suggests that the levels were below our limit of detection (~1 copy per 1,000 ng of input RNA). Finally, the IVRA assay can both overestimate and underestimate the true levels of infectious HIV RNA (33). However, since we compared time points longitudinally, this limitation does not necessarily affect our conclusions.

Of note, no amplification failures were observed at T1 for the HIV Psi and RRE DNA sequences, which detect intact HIV DNA. However, in individuals 8060 and 8036 at T2/T3, the Psi DNA was not detected (Fig. S13A and B). Given that these individuals had detectable but very low Psi levels at T1, it could be that the inability to detect Psi at T2 is the result of the low infection frequency relative to our limit of detection (~1 copy per 1,500 ng of input DNA). Likewise, we were unable to detect HIV Psi and/or RRE RNA sequences in individuals 8060, 8036, 8019, 8061, and 8053 at T1, T2, and/or T3 (Fig. S13D). However, for each of these HIV RNA samples, the corresponding sequence (Psi or RRE) was detected in the HIV DNA (using the same primers/probes) from the same cells. Thus, the lack of detection of these HIV RNA regions is likely not due to amplification failure but rather to very low levels of Psi and/or RRE RNA (<1 copy per 600 ng of input RNA). Lastly, it should be noted that the median fold decline (T1/T2), the ratios of one HIV transcript to another, and the ratios of HIV RNA/DNA could not be calculated when the denominator was 0, so that we had no data from some individuals and a reduced power for some comparisons.

In conclusion, we found evidence suggesting that many proviruses (including ~90% of intact proviruses) have blocks to HIV transcription before ART and that that different HIV transcripts decline at different rates on ART. Completed (polyadenylated) HIV transcripts decayed faster than initiated or 5′-elongated HIV transcripts, and intact HIV RNA decayed faster than defective HIV RNA. Since ART does not target HIV transcription, our findings suggest differences in the degree to which various HIV transcripts stimulate immune responses, leading to differences in the life span and/or immune clearance of various HIV-transcribing cell populations.

## MATERIALS AND METHODS

### Study participants

Blood samples were obtained from participants enrolled in the UCSF Treat Acute HIV Cohort. Peripheral blood mononuclear cells (PBMCs) were isolated before ART initiation (T1) and after 6 months (T2) and 1 year (T3) of suppressive ART. Samples were obtained from 16 participants at both T1 and T2, with T3 samples available from 11 of these participants.

### Nucleic acid isolation

Cryovials containing 10 × 10^6^ PBMCs were thawed in a 37°C water bath, diluted in phosphate-buffered saline with 2% fetal bovine serum, and centrifuged at 250 g for 5 min to pellet cells. CD4+ T cells were isolated from 2-4 vials using the EasySep Human CD4+ T Cell Enrichment Kit (StemCell Technologies). Total cellular DNA and RNA were extracted in parallel using Trireagent (Molecular Research Center) according to the manufacturer’s instructions. RNA and DNA concentrations were measured using UV spectrophotometry (NanoDrop One, Thermo Fisher).

### Quantification of HIV DNA by ddPCR

Levels of different HIV DNA regions, including the U3–U5 LTR, TAR, R-U5-pre-Gag (LLTR), and Pol regions, were quantified using ddPCR as described previously (18). HIV DNA copies were normalized to cell numbers according to the mass of DNA input per well (calculated from the DNA concentration and input volume) (62). The IPDA (duplex ddPCR for the HIV Psi and RRE regions) was performed as described previously (28, 63). Each sample was tested in at least two replicate ddPCR wells with a maximum DNA input of 750 ng per well.

### Reverse transcription and quantification of HIV RNA

Total initiated HIV (TAR) transcripts were quantified by a three-step polyadenylation-RT-ddPCR as described previously (18, 64). HIV 5′-elongated (LLTR), mid-transcribed (Pol), completed (PolyA), and multiply spliced (TatRev) transcripts were quantified by a two-step RT-ddPCR as previously described (18). HIV RNA copies were normalized to total cellular transcription (1 µg of RNA, corresponding to about 10^6^ cells) according to the RNA concentration, the volumes used in the RT, and the fraction going into each ddPCR well. The IVRA (duplex assay for the HIV Psi and RRE transcripts) was performed using a one-step dd-RT-PCR as described previously (33). Each sample was tested in at least two replicate ddPCR wells with a maximum RNA input of 300 ng per well. HIV RNA copies were normalized to cell numbers according to the mass of RNA input per well (calculated from the RNA concentration and input volume) (62).

### Statistical analysis

The Wilcoxon signed-rank test was used to compare levels of different HIV DNA regions, HIV transcripts, and ratios (HIV RNA/RNA or HIV RNA/DNA) within time points and between time points. Wells with no positive droplets were assigned a value of 0 to calculate the median and *P* values (18, 27). All statistics were performed using GraphPad Prism (version 9.5.1). *P* values that were corrected for multiple comparisons using the Benjamini–Hochberg method are outlined in Table S1.

## Data Availability

All data are available in the article and supplemental material or from the corresponding author upon request.

## SUPPLEMENTAL MATERIAL

Supplemental material jvi.01431-24-s0001.pdf Figures S1 to S13; Table S1.

ASM does not own the copyrights to Supplemental Material that may be linked to, or accessed through, an article. The authors have granted ASM a non-exclusive, world-wide license to publish the Supplemental Material files. Please contact the corresponding author directly for reuse.
