# Supplementary material for: Longitudinal changes in the transcriptionally active and intact HIV reservoir after starting ART during acute infection
Source: J Virol. 2025 Feb 5;99(3):e01431-24. doi: 10.1128/jvi.01431-24 (PMC11915860; doi:10.1128/jvi.01431-24)
Supplement: Supplemental material — Figures S1 to S13; Table S1. [file jvi.01431-24-s0001.pdf]

# Supplementary material

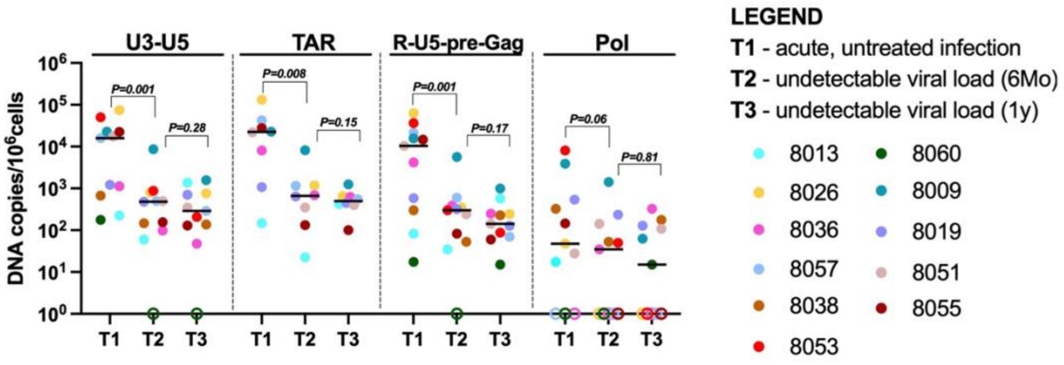


**Figure S1**. **No significant decreases in the HIV DNA levels between six months and 1 year of ART.** Total cellular DNA was extracted and the levels of U3-U5 (RdTh), TAR, R-U5-pre-Gag (LLTR), and Pol HIV DNA were measured by ddPCR before (T1) and after 6 months (T2) and 1 year (T3) of suppressive ART. The levels of each HIV DNA region were expressed as the number of copies per $10^6$ cells (assuming 1 μg of total DNA corresponds to 160,000 cells). Horizontal lines indicate medians, different colors indicate individual study participants, and open circles indicate undetectable values. P-values (two-tailed) were calculated using the Wilcoxon signed rank test.

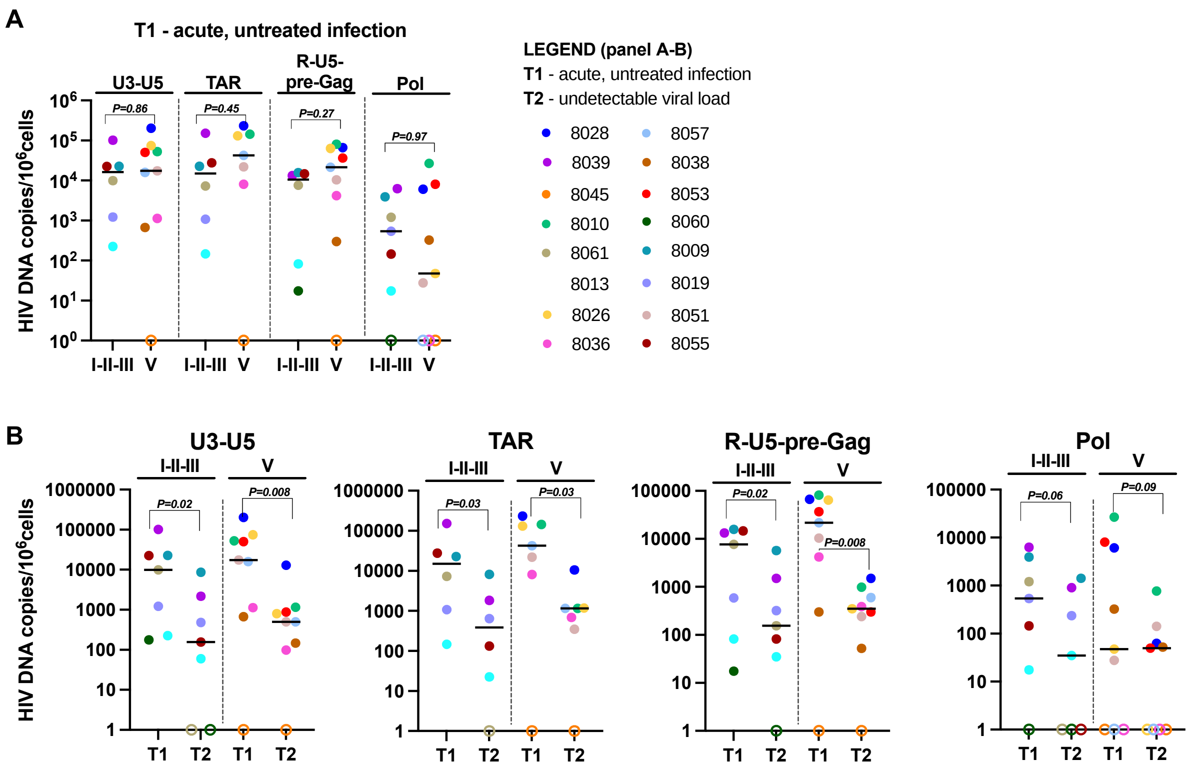


**Figure S2**. **Comparable HIV DNA levels across different Fiebig stages.** Total cellular DNA was extracted and the levels of U3-U5 (RdTh), TAR, R-U5-pre-Gag (LLTR), and Pol HIV DNA were measured by ddPCR across individuals from different Fiebig stages. The HIV DNA levels are shown **A)** before (T1) suppressive ART and **B)** before and after 6 months (T2) of ART. **A, B)** The levels of each HIV DNA region were expressed as the number of copies per $10^6$ cells (assuming 1 µg of total DNA corresponds to 160,000 cells). Horizontal lines indicate medians, different colors indicate individual study participants, and open circles indicate undetectable values. P-values (two-tailed) were calculated using the **A)** Mann-Whitney test and **B)** Wilcoxon signed rank test.

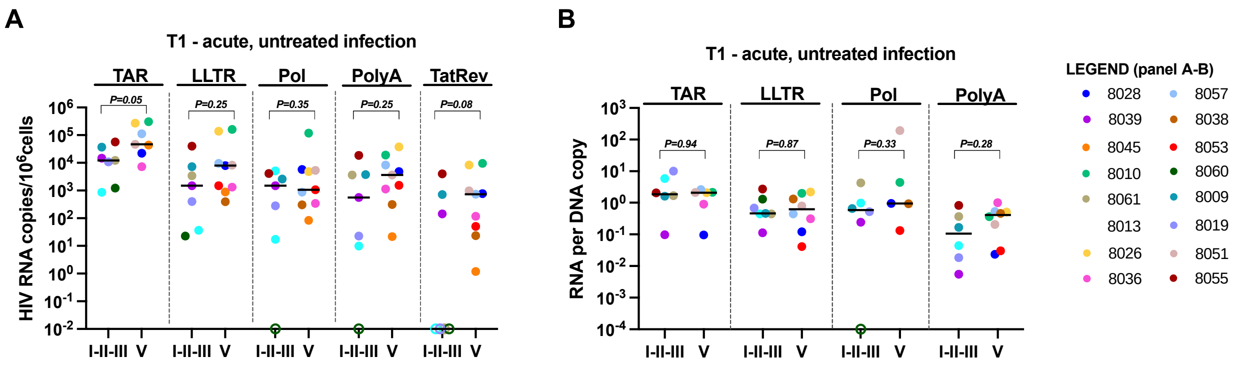


**Figure S3. Lower levels of initiated (TAR) and multiply spiced (TatRev) HIV RNA in individuals treated earlier.** Total cell-associated RNA was extracted and the progression through different stages of HIV transcription was quantified by RT-ddPCR across individuals from different Fiebig stages. **A)** The levels of initiated (TAR), 5' elongated (LLTR), mid-transcribed/unspliced (Pol), completed (PolyA), and multiply spliced (TatRev) HIV transcripts were measured and expressed as the number of copies per $10^6$ cells (assuming 1 μg of total RNA corresponds to $10^6$ cells). **B)** The levels of each HIV transcript were normalized to the corresponding HIV DNA to account for differences in infection frequency and proviral mutations. **A, B)** Horizontal lines indicate medians, different colors indicate individual study participants, and open circles indicate undetectable values. P-values (two-tailed) were calculated using the Mann-Whitney test.

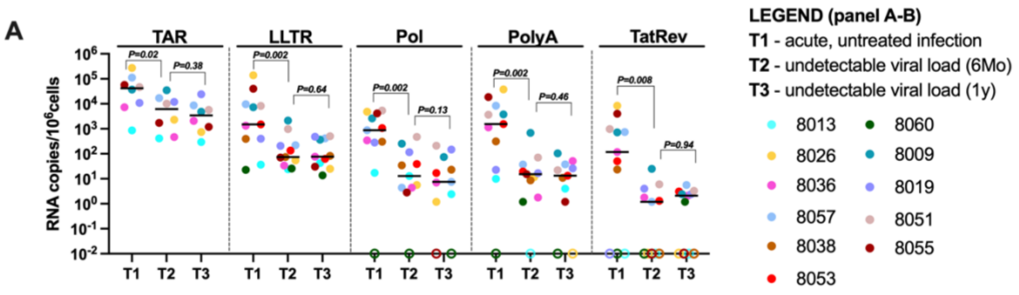


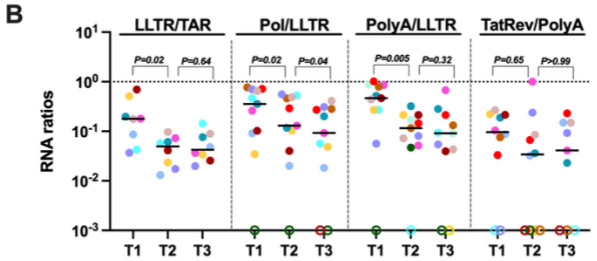


| Transcription stage | Ratio | T1 | T2 | T3 |
|---|---|---|---|---|
| elongation | LLTR/TAR | 0.18 | 0.05 | 0.04 |
| mid-transcription | Pol/LLTR | 0.35 | 0.13 | 0.09 |
| completion | PolyA/LLTR | 0.47 | 0.12 | 0.09 |
| splicing | TatRev/PolyA | 0.10 | 0.03 | 0.04 |

**Figure S4**. **No significant decreases in HIV RNA transcripts between six months and 1 year of ART.** **A)** Total cell-associated RNA was extracted and the progression through different stages of HIV transcription was quantified by RT-ddPCR measuring the levels of initiated (TAR), 5’ elongated (LLTR), mid-transcribed/unspliced (Pol), completed (PolyA), and multiply spliced (TatRev) HIV transcripts before (T1) and after 6 months (T2) and 1 year (T3) of suppressive ART. The levels of each HIV transcript were expressed as the number of copies per $10^6$ cells (assuming 1 µg of total RNA corresponds to $10^6$ cells). **B)** The ratios of one HIV RNA to another to evaluate the progression through HIV transcriptional elongation, mid-transcription, completion, and splicing before (T1) and after 6 months (T2) and 1 year (T3) of suppressive ART. Ratios are independent of effects at prior stages of HIV transcription, and independent of infection frequency or normalization to cell numbers. Shown are the proportion of i) all HIV transcripts that were elongated [LLTR/TAR]; ii) elongated HIV transcripts that were mid-transcribed [Pol/LLTR] or iii) completed [PolyA/LLTR]; and iv) completed transcripts that were multiply spliced [TatRev/PolyA]. **A, B)** Horizontal lines indicate medians, different colors indicate individual study participants, and open circles indicate undetectable values. P-values (two-tailed) were calculated using the Wilcoxon signed rank test.

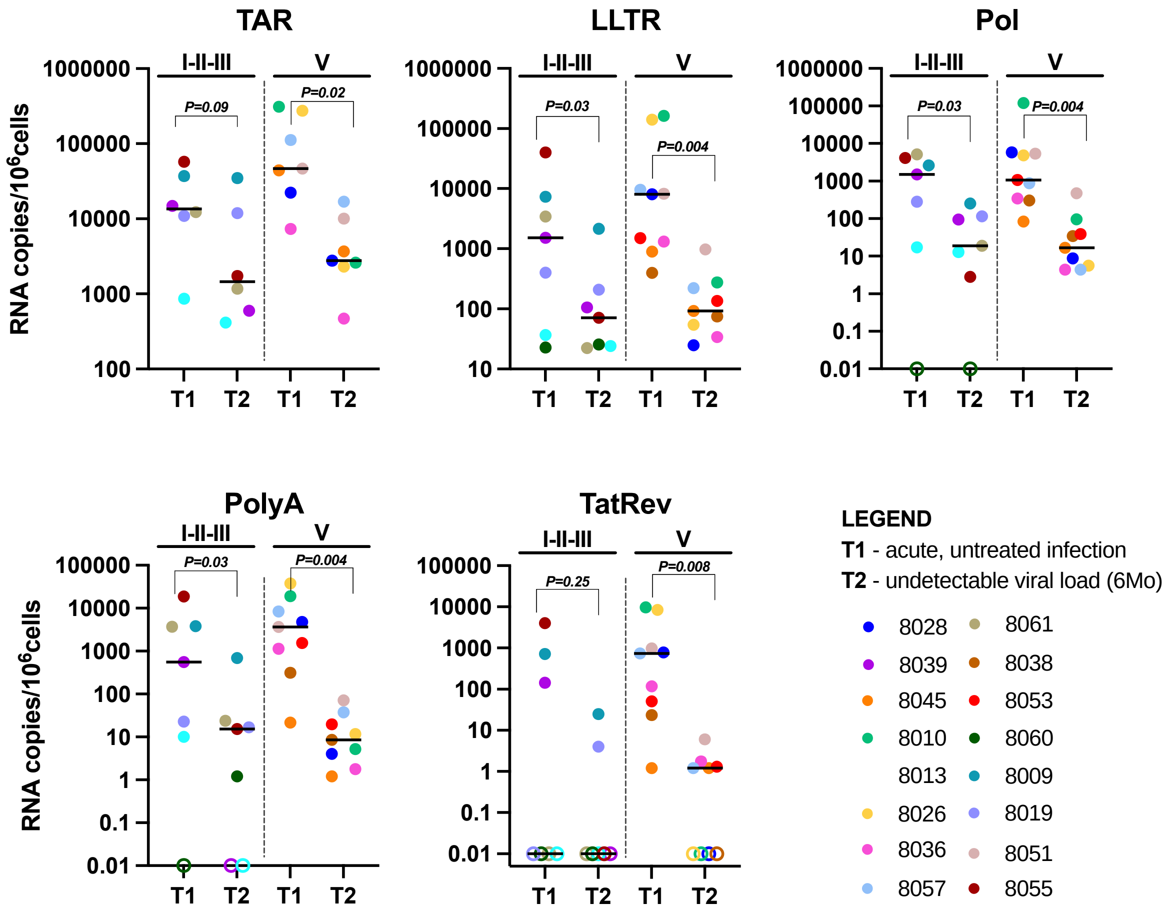


**Figure S5. Decreases in HIV RNA in individuals treated during Fiebig stages I-III compared to stage V.** Total cell-associated RNA was extracted and the progression through different stages of HIV transcription was quantified by RT-ddPCR across individuals from different Fiebig stages. The levels of initiated (TAR), 5’ elongated (LLTR), mid-transcribed/unspliced (Pol), completed (PolyA), and multiply spliced (TatRev) HIV transcripts were measured before (T1) and after 6 months (T2) of suppressive ART and expressed as the number of copies per $10^6$ cells (assuming 1 μg of total RNA corresponds to $10^6$ cells). Horizontal lines indicate medians, different colors indicate individual study participants, and open circles indicate undetectable values. P-values (two-tailed) were calculated using the Wilcoxon signed rank test.

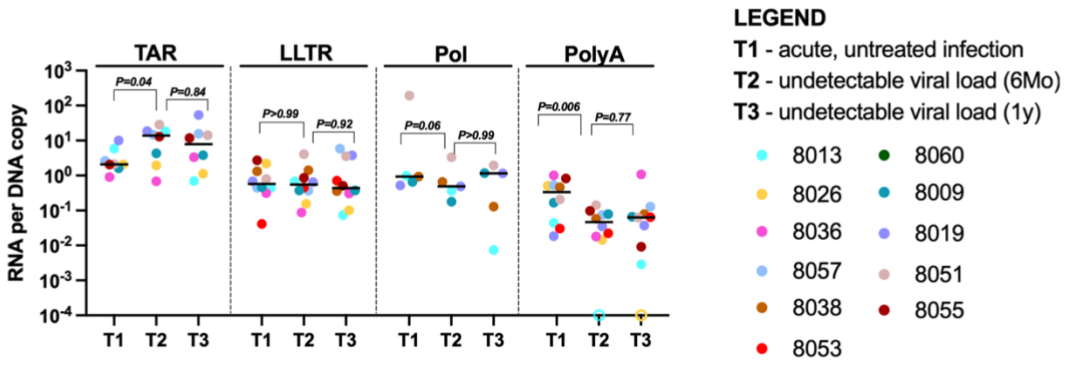


**Figure S6. No significant decreases in HIV RNA per DNA between six months and 1 year of ART.** The levels of each HIV transcript were normalized to the corresponding HIV DNA to account for differences in infection frequency and proviral mutations. HIV transcripts per provirus are shown before (T1) and after 6 months (T2) and 1 year (T3) of suppressive ART. Horizontal lines indicate medians, different colors indicate individual study participants, and open circles indicate undetectable values. P-values (two-tailed) were calculated using the Wilcoxon signed rank test.

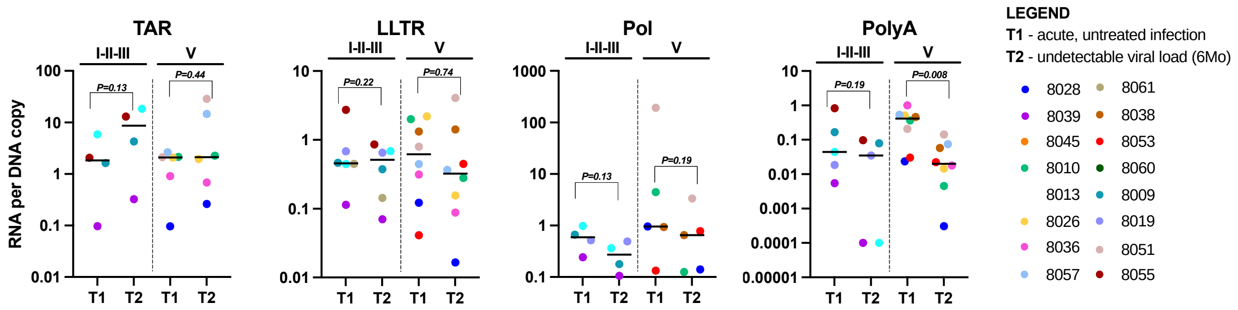


**Figure S7. Decreases in completed (PolyA) HIV RNA per provirus in individuals treated during Fiebig stages I-III compared to stage V.** The levels of each HIV transcript were normalized to the corresponding HIV DNA to account for differences in infection frequency and proviral mutations. The HIV transcripts per DNA copy are shown before (T1) and after 6 months of suppressive ART (T2). Horizontal lines indicate medians, different colors indicate individual study participants. P-values (two-tailed) were calculated using the Wilcoxon signed rank test.

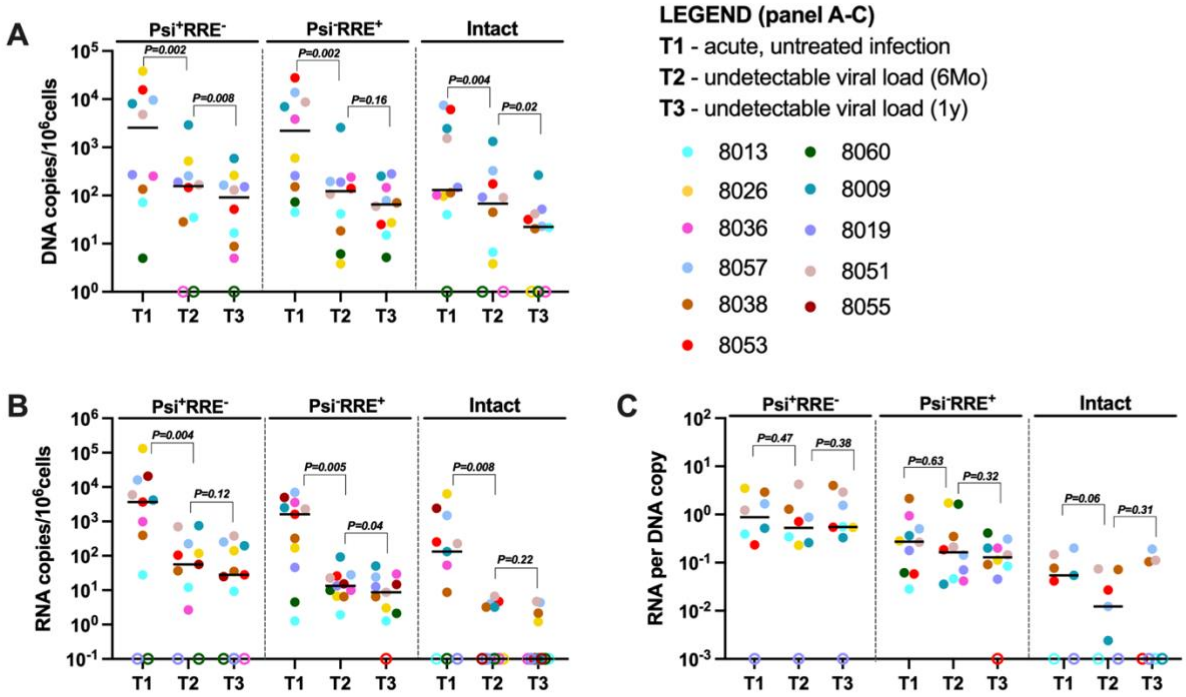


**Figure S8**. **Small decrease in intact HIV DNA but not intact RNA between six months and 1 year of ART. A)** Total cellular DNA was extracted and the levels of 3'defective (Psi+RRE-), 5' defective (Psi-RRE+), and intact (Psi+RRE+) HIV DNA were measured using the intact proviral DNA assay (IPDA) before (T1) and after 6 months (T2) and 1 year (T3) of suppressive ART. The levels of each HIV DNA region were expressed as the number of copies per $10^6$ cells (assuming 1 μg of total DNA corresponds to 160,000 cells). **B)** Total cell-associated RNA was extracted and the levels of 3'defective (Psi+RRE-), 5' defective (Psi-RRE+), and intact (Psi+RRE+) HIV RNA were measured using the intact viral RNA assay (IVRA) before (T1) and after 6 months (T2) and 1 year (T3) of suppressive ART. The levels of each HIV transcript were expressed as the number of copies per $10^6$ cells (assuming 1 μg of total RNA corresponds to $10^6$ cells). **C)** The levels of each HIV transcript (3' defective, 5' defective and intact) were normalized to the corresponding HIV DNA to account for differences in infection frequency and proviral mutations. HIV transcripts per provirus are shown before (T1) and after 6 months and 1 year (T3) of suppressive ART. **A-C)** Horizontal lines indicate medians, different colors indicate individual study participants, and

open circles indicate undetectable values. P-values (two-tailed) were calculated using the Wilcoxon signed rank test.

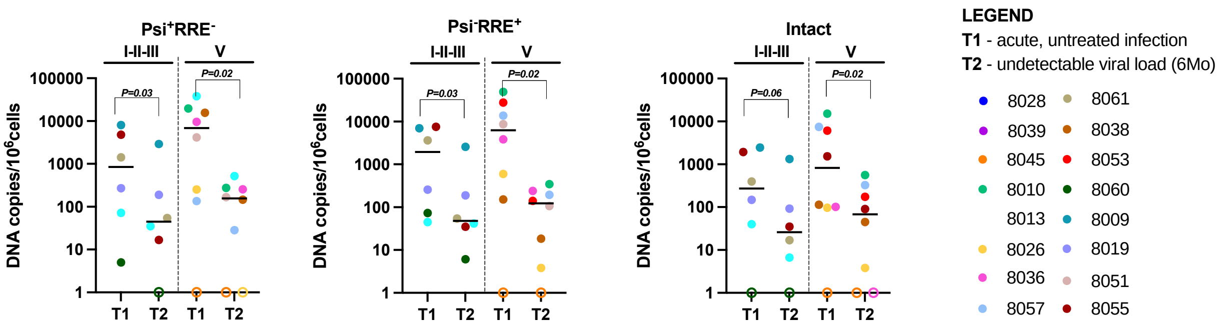


**Figure S9. Comparable reductions in intact and defective HIV DNA across different Fiebig stages.** Total cellular DNA was extracted and the levels of 3'defective (Psi+RRE-), 5' defective (Psi-RRE+), and intact (Psi+RRE+) HIV DNA were measured across individuals from different Fiebig stages using the intact proviral DNA assay (IPDA). The HIV DNA levels were measured before (T1) and after 6 months of suppressive ART (T2) and expressed as the number of copies per $10^6$ cells (assuming 1 µg of total DNA corresponds to 160,000 cells). Horizontal lines indicate medians, different colors indicate individual study participants, and open circles indicate undetectable values. P-values (two-tailed) were calculated using the Wilcoxon signed rank test.

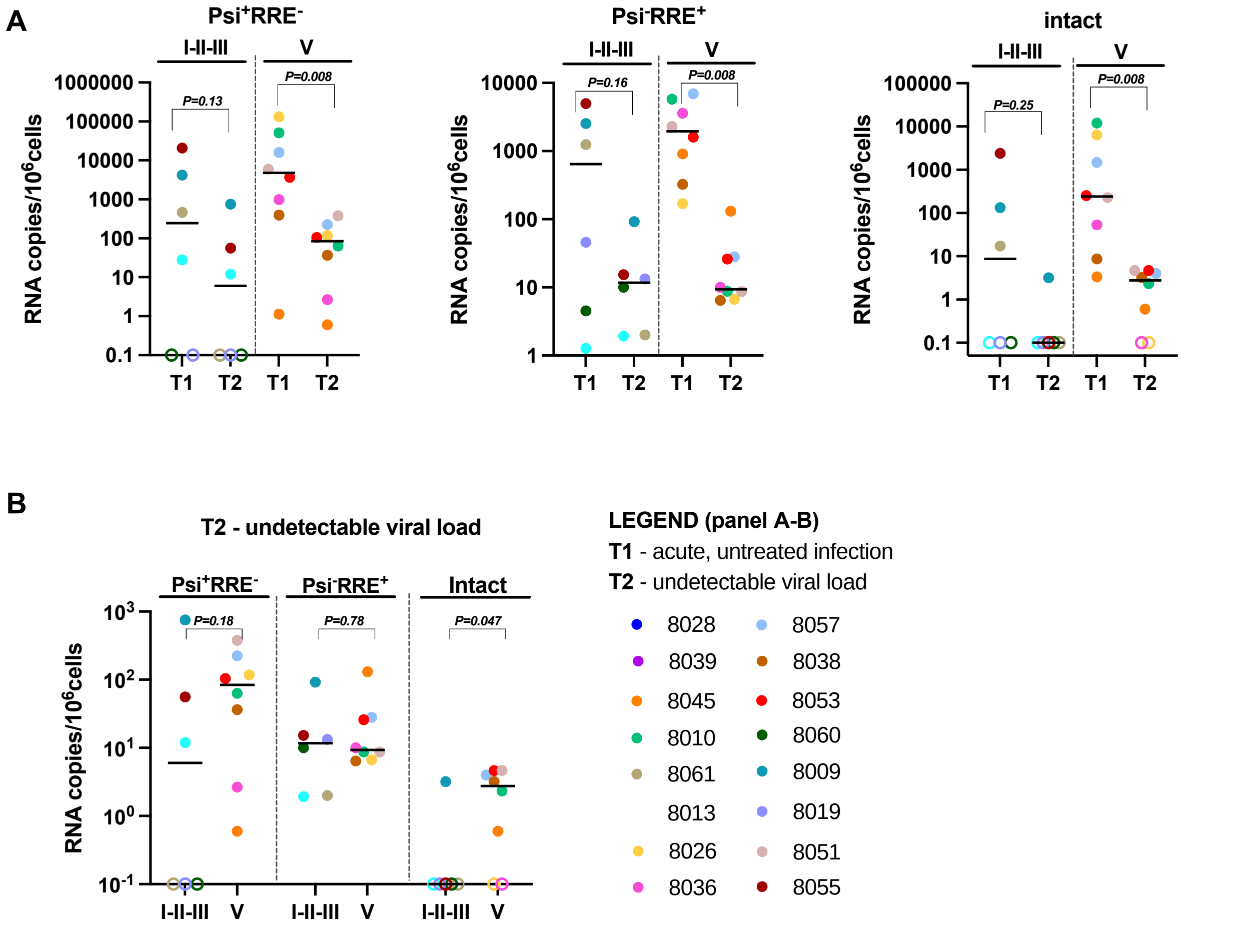


**Figure S10. Decreases in intact and defective HIV RNA in individuals treated during Fiebig stages I-III compared to stage V.** Total cell-associated RNA was extracted and the levels of 3'defective (Psi+RRE-), 5' defective (Psi-RRE+), and intact (Psi+RRE+) HIV RNA were measured across individuals from different Fiebig stages using the intact viral RNA assay (IVRA). The HIV RNA levels are shown **A)** before (T1) and after 6 months of suppressive ART (T2) and **B)** after 6 months of suppressive ART. **A, B)** The levels of each HIV transcript were expressed as the number of copies per $10^6$ cells (assuming 1 µg of total RNA corresponds to $10^6$ cells). Horizontal lines indicate medians, different colors indicate individual study participants, and open circles indicate undetectable values. P-values (two-tailed) were calculated using the **A)** Wilcoxon signed rank test and **B)** Mann-Whitney test.

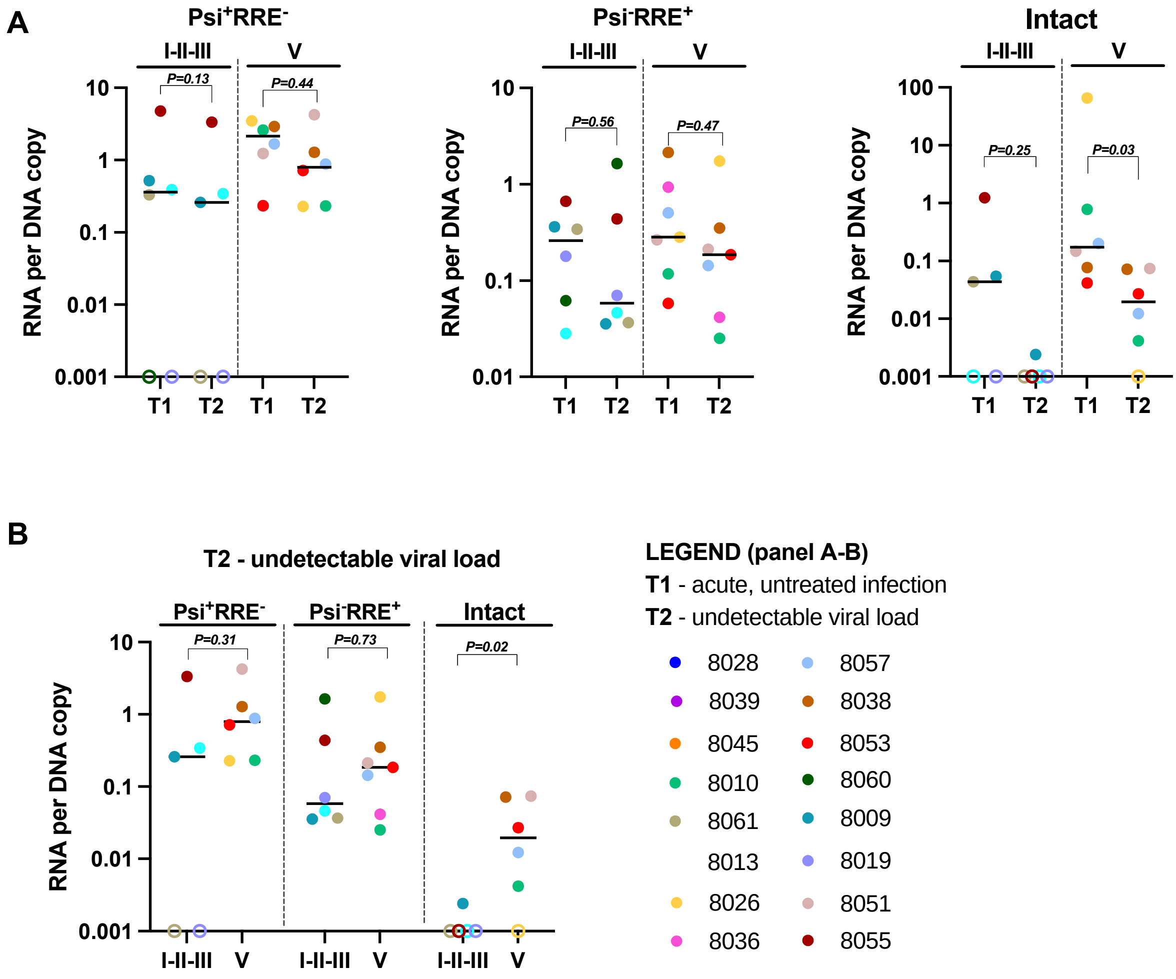


**Figure S11. Decrease in intact HIV RNA per intact provirus in individuals treated during Fiebig stages I-III compared to stage V.** The levels of each HIV transcript were normalized to the corresponding HIV DNA to account for differences in infection frequency and proviral mutations. The HIV transcripts per DNA copy are shown **A)** before (T1) and after 6 months of suppressive ART (T2) and **B)** after 6 months of suppressive ART. **A, B)** Horizontal lines indicate medians, different colors indicate individual study participants, and open circles indicate undetectable values. P-values (two-tailed) were calculated using the **A)** Wilcoxon signed rank test and **B)** Mann-Whitney test.

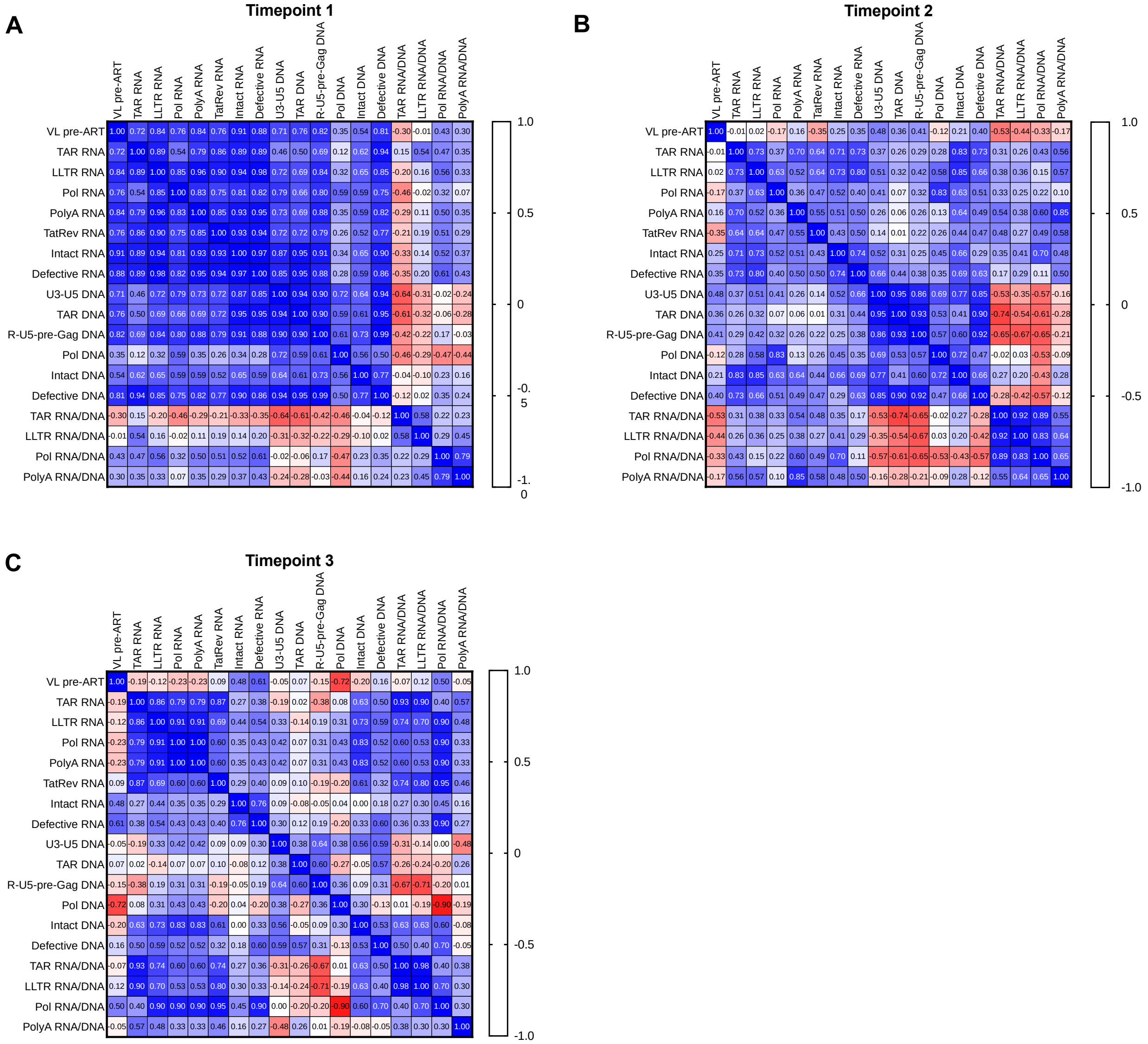


**Figure S12. Spearman correlation analysis between pre-ART viral load (T1) and HIV DNA and RNA measures at T1-T3.** Shown are correlation matrices comparing the pre-ART viral load and levels of different HIV transcripts, levels of different HIV DNA regions, and ratios of HIV RNA to the corresponding HIV DNA (average transcription per provirus) at T1 (A), T2 (B), and T3 (C). Numbers indicate r values from Spearman correlations. White numbers denote two-tailed Spearman P values <0.05, while black numbers indicate P>0.05. Results were not corrected for multiple comparisons. The background of each box has been colored according to r value, as shown in the color scale at right, with darker blue indicating more positive r values and darker red indicating more negative r values.

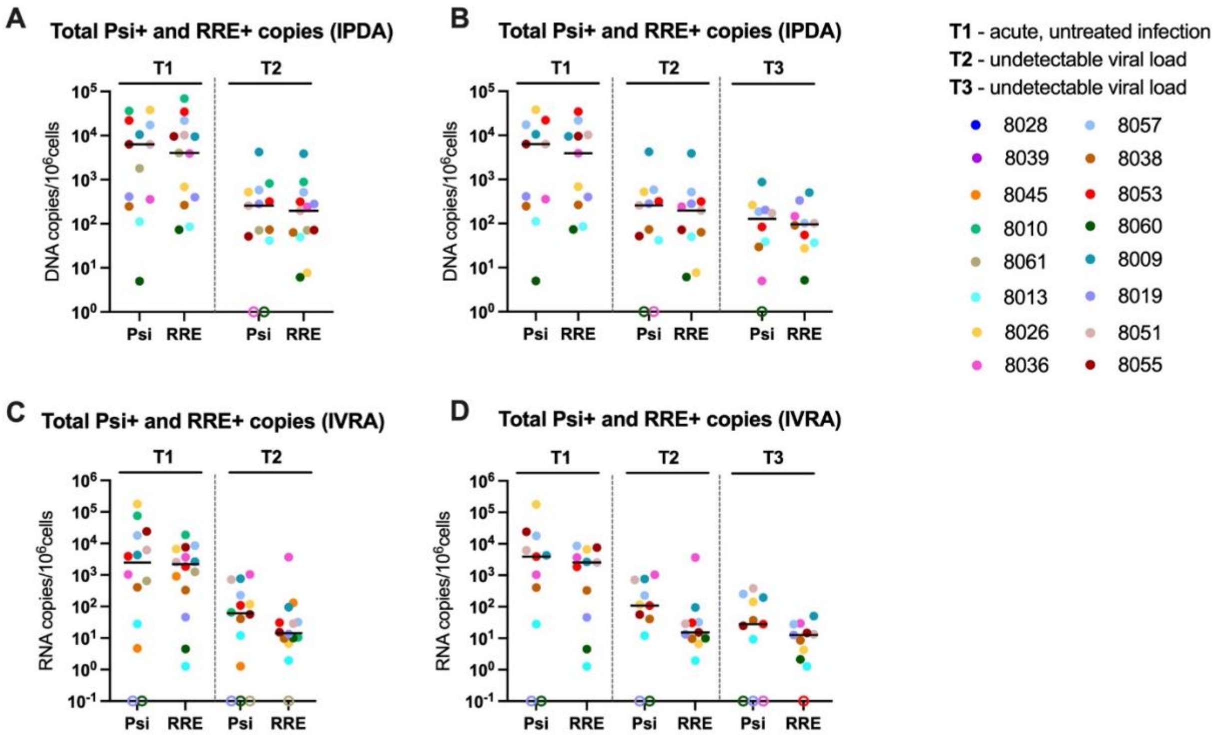


**Figure S13. No evidence of apparent amplification failures. A, B)** Total cellular DNA was extracted and the total levels of the HIV Packaging Signal [Psi] DNA (Psi+RRE+, Psi+RRE-) and HIV Rev Response Element [RRE] DNA (Psi+RRE+, Psi-RRE+) were measured using the intact proviral DNA assay (IPDA) before (T1) and **A)** after 6 months (T2) and **B)** 1 year (T3) of suppressive ART. The levels of each HIV DNA region were expressed as the number of copies per $10^6$ cells (assuming 1 μg of total DNA corresponds to 160,000 cells). Horizontal lines indicate medians, and different colors indicate individual study participants. **C, D)** Total cell-associated RNA was extracted and the total levels of the HIV Packaging Signal [Psi] RNA (Psi+RRE+, Psi+RRE-) and HIV Rev Response Element [RRE] RNA (Psi+RRE+, Psi-RRE+) were measured using the intact viral RNA assay (IVRA) before (T1) and **C)** after 6 months (T2) and **D)** 1 year (T3) of suppressive ART. The levels of each HIV transcript were expressed as the number of copies per $10^6$ cells (assuming 1 μg of total RNA corresponds to $10^6$ cells). Horizontal lines indicate medians, different colors indicate individual study participants, and open circles indicate undetectable values.

| | | P-value | Corrected P value |
|---|---|---|---|
| 1 | TAR vs PolyA | 0.0002 | 0.005555556 |
| 2 | LLTR vs PolyA | 0.0067 | 0.011111111 |
| 3 | LLTR vs TatRev | 0.0342 | 0.016666667 |
| 4 | LLTR vs Pol | 0.0554 | 0.022222222 |
| 5 | TAR vs LLTR | 0.0681 | 0.027777778 |
| 6 | TAR vs TatRev | 0.084 | 0.033333333 |
| 7 | Pol vs TatRev | 0.0923 | 0.038888889 |
| 8 | TAR vs Pol | 0.0942 | 0.044444444 |
| 9 | PolyA vs TatRev | 0.5195 | 0.05 |

**Table S1. P-values corrected for multiple comparisons using the Benjamini–Hochberg method.** Each individual P value was compared to its Benjamini-Hochberg critical value, (i/m)Q, where i is the rank, m is the total number of tests, and Q is the false discovery rate (0.05). The largest $P$-value that has $P<(i/m)$Q is significant, and all of the $P$-values smaller than it are also significant, even the ones that are not less than their Benjamini-Hochberg critical value.
